# Shotgun proteomics reveals physiological response to ocean acidification in *Crassostrea gigas*

**DOI:** 10.1186/1471-2164-15-951

**Published:** 2014-11-03

**Authors:** Emma Timmins-Schiffman, William D Coffey, Wilber Hua, Brook L Nunn, Gary H Dickinson, Steven B Roberts

**Affiliations:** School of Aquatic and Fishery Sciences, University of Washington, Box 355020, Seattle, WA 98195 USA; Department of Biology, The College of New Jersey, 2000 Pennington Road, Ewing, NJ 08628 USA; Genome Sciences, University of Washington, Box 355065, Seattle, WA 98195 USA

**Keywords:** Shell deposition, Fatty acid, Proteomics, Ocean acidification, Shell mechanical properties, Pacific oyster

## Abstract

**Background:**

Ocean acidification as a result of increased anthropogenic CO_2_ emissions is occurring in marine and estuarine environments worldwide. The coastal ocean experiences additional daily and seasonal fluctuations in pH that can be lower than projected end-of-century open ocean pH reductions. In order to assess the impact of ocean acidification on marine invertebrates, Pacific oysters (*Crassostrea gigas*) were exposed to one of four different *p* CO_2_ levels for four weeks: 400 μatm (pH 8.0), 800 μatm (pH 7.7), 1000 μatm (pH 7.6), or 2800 μatm (pH 7.3).

**Results:**

At the end of the four week exposure period, oysters in all four *p* CO_2_ environments deposited new shell, but growth rate was not different among the treatments. However, micromechanical properties of the new shell were compromised by elevated *p* CO_2_. Elevated *p* CO_2_ affected neither whole body fatty acid composition, nor glycogen content, nor mortality rate associated with acute heat shock. Shotgun proteomics revealed that several physiological pathways were significantly affected by ocean acidification, including antioxidant response, carbohydrate metabolism, and transcription and translation. Additionally, the proteomic response to a second stress differed with *p* CO_2_, with numerous processes significantly affected by mechanical stimulation at high versus low *p* CO_2_ (all proteomics data are available in the ProteomeXchange under the identifier PXD000835).

**Conclusions:**

Oyster physiology is significantly altered by exposure to elevated *p* CO_2_, indicating changes in energy resource use. This is especially apparent in the assessment of the effects of *p* CO_2_ on the proteomic response to a second stress. The altered stress response illustrates that ocean acidification may impact how oysters respond to other changes in their environment. These data contribute to an integrative view of the effects of ocean acidification on oysters as well as physiological trade-offs during environmental stress.

**Electronic supplementary material:**

The online version of this article (doi:10.1186/1471-2164-15-951) contains supplementary material, which is available to authorized users.

## Background

Current measurements of surface ocean pH have revealed decreases that are in accordance with modeled predictions of a pH decline of at least 0.3 units corresponding to atmospheric *p* CO_2_ of 650-970 ppm by the year 2100 [1–6]. The coastal ocean, home to productive fisheries and diverse ecosystems, may see even greater changes in pH due to natural processes (i.e. hydrography, freshwater input, and biological activity) [7–11] and a plethora of anthropogenic effects (i.e. deforestation, agriculture, mining, increasing population sizes [7]). Although some species that live in the coastal ocean show a degree of adaptation to variable pH [12, 13] sessile invertebrates are sensitive to acute low pH exposures across life stages. In bivalves, low pH results in significant changes to larval development (e.g. [14]), reduced shell deposition in most species (e.g. [15, 16]), decreased integrity of the shell [17, 18] and weakened attachment of byssal threads [19]. In addition to phenotypic impacts, elevated *p* CO_2_ can result in significant shifts in marine invertebrate metabolism and resource utilization (e.g. [20]).

The Pacific oyster, *Crassostrea gigas*, is a marine invertebrate that has been well studied in terms of its response to ocean acidification. Larvae experience developmental delay and shell malformations in response to low pH [14, 21–24]. Adults are also affected negatively by ocean acidification. Fertilization success is reduced at moderately elevated *p* CO_2_ (600 μatm) [23]. Calcification rates for adult *C. gigas* decrease linearly with increasing *p* CO_2_[15]. Reduced pH also alters response to other environmental variables. The standard metabolic rate of Pacific oysters at low pH was significantly elevated in response to increasing temperature compared to oysters at ambient pH [25]. Such studies illustrate that ocean acidification causes profound physiological changes in *C. gigas* that may have long-term consequences on fitness.

To examine the underlying processes associated with the biological impacts of ocean acidification on marine invertebrates, the current study takes an integrative approach in examining the response of adult oysters from alterations in protein abundance to shell deposition rates, shell micromechanical structure, tissue glycogen and fatty acid contents, mortality in response to acute heat shock, and proteomic response to mechanical stress. Oysters were exposed to one of four *p* CO_2_ levels (400, 800, 1000, or 2800 μatm) for one month. The *p* CO_2_ values represent approximate current-day surface ocean *p* CO_2_ (400 μatm) and three elevated values reflecting potential end-of-century scenarios as well as *p* CO_2_ variation that is currently experienced in the nearshore environment. At the end of one month the impacts of elevated *p* CO_2_ on shell growth, shell micromechanical properties, lipid metabolism, glycogen metabolism, response to acute heat shock, and response to mechanical stress were assessed.

Acute heat shock and mechanical stress represent a test of the mechanistic limits of the stress response and a simulation of ecological stress, respectively. Whereas the oysters may not experience a heat shock in their natural environment that attains the temperature of the one we applied, the stressor serves as an assessment of the mortality response to an intense environmental change. The mechanical stress stimulates a more subtle, yet significant, stress response that is physiologically similar to the oyster’s response to other relevant environmental stresses [26, 27].

By taking this integrative approach, these data highlight the complex nature of phenotypic impacts of ocean acidification, while at the same time uncovering the less accessible underlying physiological processes. The latter was made possible by the use of shotgun proteomics, applied for the first time in an investigation of the effects of ocean acidification. Shotgun proteomics is a powerful non-biased approach in the investigation of biological responses, which also offers insight into underlying mechanisms that could lead to phenotypic effects. Together these data demonstrate the scope of effects that ocean acidification can have on a marine invertebrate.

## Results & discussion

Ocean acidification is an on-going and global scale phenomenon that has been shown to negatively impact most taxonomic groups. A large number of studies have characterized these negative impacts across species and life stages (e.g. [28, 29]). With the goal of achieving a better understanding of the effects of ocean acidification and responses to additional acute stressors across multiple physiological processes, we employed an integrative approach, combining analyses of shell micromechanical properties, fatty acids, glycogen concentration, and proteomics. Our findings illustrate the value of using complementary approaches to explore interactions and trade-offs among different fundamental processes during environmental stress.

### Seawater chemistry analysis

The *p* CO_2_ levels for the four different treatments remained consistent throughout the one month experiment (Table 1). Average pH (± s.d.) for treatments as measured by the DuraFET probe were 8.02 ± 0.02, 7.73 ± 0.04, 7.63 ± 0.10, and 7.29 ± 0.10 for the 400, 800, 1000, and 2800 μatm treatments, respectively. Spectrophotometric pH corroborated the DuraFET measurements (spectrophotometric pH data not shown). The *p* CO_2_ in containers with oysters was approximately 40 μatm higher than the source water *p* CO_2_, except for the 2800 μatm treatment where it was approximately 75 μatm lower than the source water. Total alkalinity was 9% higher in the chambers (data not shown) compared to the source water for 400 μatm, 10% higher at 800 μatm, 13% higher at 1000 μatm, and 3% lower at 2800 μatm. Calcite was undersaturated (Ω_c_ <1.0) only at the highest *p* CO_2_ level and aragonite was undersaturated at the two highest *p* CO_2_ levels (1000 μatm and 2800 μatm).

**Table 1:** **Water chemistry summary data**

Treatment (ppm CO _2_ )	pH	Temperature (°C)	Salinity	A _T_ (μmol/kg)	Source water pCO _2_ (μatm)	Oyster container pCO _2_ (μatm)	Ω (calcite)	Ω (aragonite)	CO _3_^2-^ (μmol/kg)
400	8.02 ± 0.02	12.97 ± 0.41	29.9 ± 0.2	2085.4 ± 15.9	427 ± 33	464 ± 54	2.7 ± 0.1	1.7 ± 0.1	109.0 ± 5.8
800	7.73 ± 0.04	12.92 ± 0.41	29.9 ± 0.2	2086.4 ± 12.1	810 ± 61	936 ± 17	1.6 ± 0.1	1.0 ± 0.1	65.3 ± 4.3
1000	7.63 ± 0.10	12.82 ± 0.21	29.9 ± 0.3	2084.9 ± 15.3	991 ± 10	1119 ± 40	1.3 ± 0	0.8 ± 0	54.7 ± 1.2
2800	7.29 ± 0.10	12.87 ± 0.51	29.9 ± 0.2	2085.6 ± 14.9	2848 ± 603	2776 ± 603	0.5 ± 0.1	0.3 ± 0.1	22.0 ± 4.5

Mean and ± standard deviation are provided for the 29 day experiment. Salinity is an average of nineteen measurements and A_T_ was measured four times. pH and temperature values are from the continuous monitoring by the DuraFET probe. pH, temperature, salinity, and A_T_ were directly measured and all other parameters were calculated using CO_2_calc [68].

### Oyster growth

Relative growth rate (RGR) for shell mass of oysters exposed to increased *p* CO_2_, as measured by buoyant weight, was not significantly different among treatments (p >0.05) (Table 2). Shell mass changed with time across all treatments (F =6.1190, p =0.014), indicating shell growth. The lack of significant difference in growth among treatments is somewhat surprising, especially considering that calcite, the main component of adult oyster shells, was undersaturated in the 2800 μatm treatment. Over longer exposures to ocean acidification, other bivalve species have demonstrated decreased shell growth as compared to individuals held at ambient *p* CO_2_. After six weeks, mussel (*Mytilus edulis*) shell length growth was negatively impacted by elevated *p* CO_2_ of 2400 and 4000 μatm [16]. Juvenile oysters, *Crassostrea virginica*, also had relatively lower shell mass after 45 days at 1 665 μatm [30] and after a 20 week exposure at 3 523 μatm [31]. Based on these data we might expect to see differences in shell growth if exposure was continued for a longer period of time. In addition to the duration of exposure, elevated *p* CO_2_ may in fact prompt an over-compensation in terms of shell deposition. For instance, hard shell clams, *Mercenaria mercenaria*, had higher shell mass at moderately elevated *p* CO_2_ (about 800 μatm) after 16 and 21 weeks of exposure, but no significant difference in shell mass between the control treatment and highly elevated *p* CO_2_ (about 1500 μatm) was observed [18]. At least some of the variability in the effects of elevated *p* CO_2_ on shell growth is likely due to intra- and interspecies differences in population history of exposure to low pH events.

**Table 2:** **Average buoyant weight ±95% confidence intervals at start and end of experiment**

pCO _2_ (μatm)	Mass at start (grams)	Mass at 29 days (grams)
400	7.1 ± 0.5	7.8 ± 0.6
800	7.0 ± 0.5	7.6 ± 0.7
1000	7.1 ± 0.4	7.4 ± 0.7
2800	7.6 ± 0.5	8.1 ± 0.9

### Micromechanical properties

**Figure 1:**
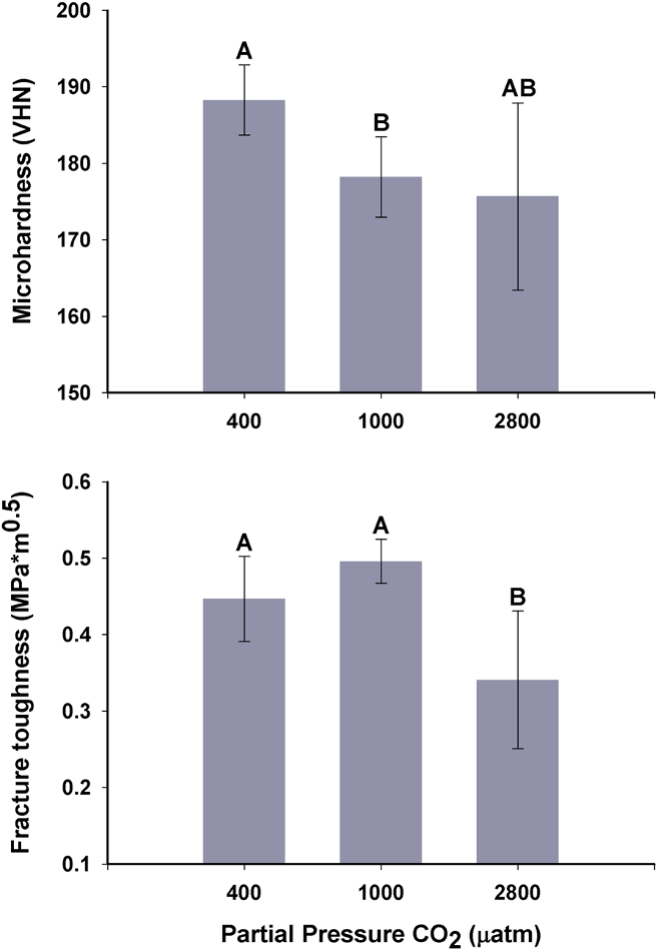
**Mean Vickers microhardness and fracture toughness of** ***C. gigas*** **shells (±95% C.I.) from 400, 1000, and 2800 μatm** ***p*** **CO**_**2**_ **, tested within the outer 3 mm of the shell’s growing edge.** Groups marked with different letters are significantly different (p <0.05; n =5-7 shells per treatment).

Micromechanical properties were tested within the outer 3 mm of the growing edge (posterior) of left shell valves for oysters in the treatments of 400, 1000, and 2800 μatm. Both Vickers microhardness and fracture toughness differed significantly among *p* CO_2_ treatments (microhardness: Welch ANOVA, p =0.014; fracture toughness: one-way ANOVA, p =0.003) (Figure 1). The microhardness of shells grown at 1000 μatm was significantly lower than that of shells grown at 400 μatm (Games-Howell: p <0.05). Shells grown at 2800 μatm showed a trend toward lower microhardness as compared to the 400 μatm control group, but this comparison was not statistically significant (Games-Howell: p =0.119). In contrast, fracture toughness was significantly lower in shells grown at 2800 μatm as compared to both the 400 and 1000 μatm treatments, but the 400 and 1000 μatm treatments did not differ (Tukey HSD: p <0.05). Representative cracks formed by micromechanical testing are shown in Additional file 1: Figure S1.

Both microhardness and fracture toughness are affected by the arrangement and dimensions of the microstructures that comprise the shell and the extent and distribution of elastic elements within the shell (i.e. the shell organic matrix) [32]. Hence, exposure to elevated *p* CO_2_ may lead to dose-dependent differences in the structure and/or composition of newly formed shell. Such changes could result either from alterations in the physiology of shell deposition or an inability to prevent dissolution and erosion of individual microstructures under varying seawater hydrochemistry. In the current study, changes to micromechanical properties were detected at both Ω_calcite_ (Ω_c_) =0.5 (2800 μatm) and at Ω_c_ =1.3 (1000 μatm). Alterations in shell growth and structure would be expected at Ω_c_ <1, however, there is increasing evidence that shell modifications also occur at Ω_c_ >1. Even when seawater is saturated with respect to calcite, bivalves experience both shell dissolution [18, 25] and changes to shell microstructure [18, 31, 33]. These results indicate that as Ω decreases the driving force towards biomineralization is reduced. Source population evolutionary history may also affect sensitivity to changes in Ω. Since calcification is an energetically intensive process [34–36], one explanation for significant effects on CaCO_3_ structures at Ω >1 is that resources for energy metabolism are being reallocated to other, non-calcification physiological processes in order to maintain homeostasis.

### Fatty acids and glycogen

**Figure 2:**
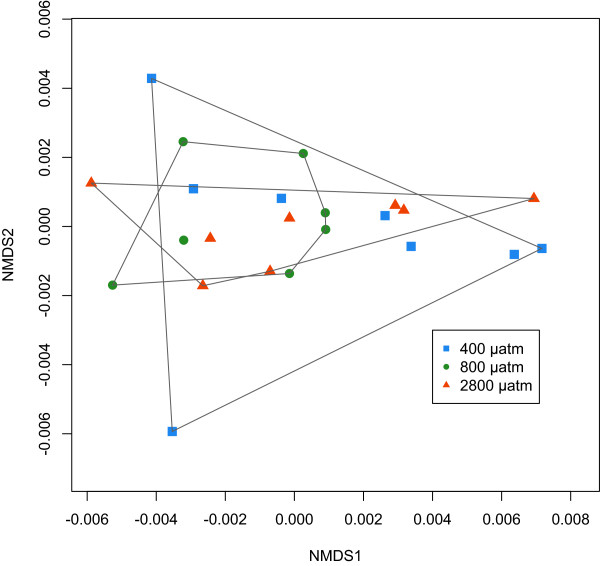
**Non-metric multidimensional scaling (NMDS) analysis of fatty acid profiles for oysters from 400, 800 and 2800 μatm** ***p*** **CO**_**2**_ **.** There are no differences in relative amounts of fatty acids among the three treatment groups.

In order to gain insight into changes in energy metabolism that could be a result of elevated *p* CO_2_, we examined fatty acid profiles and glycogen content. Fatty acid profiles did not differ among three treatments (400, 800, and 2800 μatm) (p >0.05; Figure 2). The nonmetric multidimensional scaling (NMDS) analysis shows the relative position of each oyster according to its entire fatty acid profile (i.e. oysters that are plotted close together have similar fatty acid profiles). Total fatty acid content (per milligram tissue) did not vary among treatments (p >0.05; data not shown). Twenty-one fatty acid peaks were identified in the 24 samples, which is within the range of 16-35 fatty acids found in other studies of bivalves [37–40]. Some of the more important fatty acids identified were 16:0; 18:0; 18:1n-9; 18:1n-7; 18:2n-6; α-linolenic acid (18:3n-3); arachidonic acid (20:4n-6); eicosapentaenoic acid (20:5n-3); two docosapentaenoic acids (22:5n-6 and 22:5n-3); and docosahexaenoic acid (22:6n-3). Raw and normalized fatty acid data are available in Additional file 2: Table S1.

**Figure 3:**
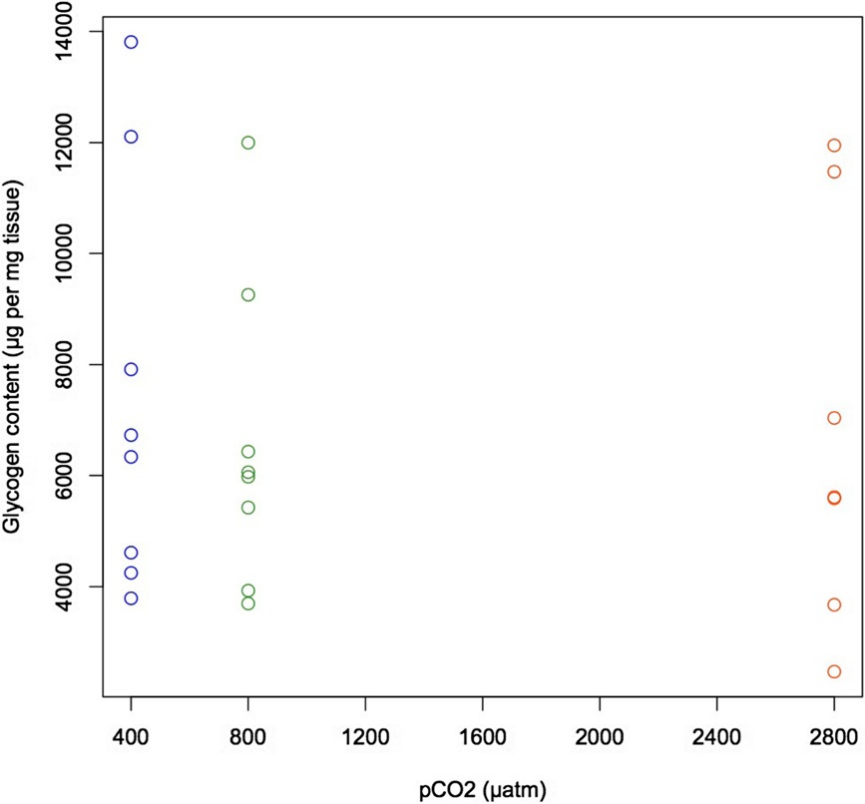
**Glycogen content (μg glycogen per mg tissue) for oysters from the** ***p*** **CO**_**2**_ **treatments of 400 (blue), 800 (green), and 2800 (orange) μatm.** There is no difference in glycogen content among treatment groups.

Glycogen content did not differ statistically among treatments (400, 800, and 2800 μatm) (p >0.05, Figure 3). However, it should be noted that the broad variance in glycogen content within treatments makes it difficult to draw a clear conclusion about the effects of *p* CO_2_. For whole body tissue, glycogen measurements ranged from 2466-13808 μg/mg of tissue with a mean (±95% confidence interval) glycogen content of 6961 ± 1406 μg/mg of tissue.

The similar relative proportions of fatty acids and glycogen content in whole body tissue among treatments suggests that extended stress of ocean acidification did not alter fundamental metabolites. Oysters and other bivalves are highly dependent on fatty acids as a main energy source, especially poly-unsaturated fatty acids [38–41]. Changes in physiological state, such as those induced by reproduction or poor nutrient supply, can alter the relative proportions of fatty acids in oyster tissue [37, 40]. Glycogen stores represent important sources of stored energy that are accumulated during the non-reproductive season and then mobilized for use as glucose during gametogenesis [42]. It is possible that an environmental stress can trigger a change in bivalve physiology and result in changes in lipid or carbohydrate metabolism and/or storage. In the case of ocean acidification, *C. gigas* is able to maintain homeostasis of both total and relative amounts of both of these energy-providing molecules by restructuring the proteome to metabolize or synthesize lipids and carbohydrates as needed. In contrast to our results, after 11 weeks of exposure to *p* CO_2_ of 800 μatm, juvenile *C. virginica* had significantly less lipid and glycogen per gram body weight than control oysters [17]. The extended stress of the 11 week exposure in Dickinson et al. [17] may have overwhelmed *C. virginica*’s ability to maintain lipid and carbohydrate homeostasis, suggesting that oyster energy metabolism may fail under consistent ocean acidification stress. There are some instances where ocean acidification did not influence lipid levels in invertebrates. For example, in larval sea urchins, despite the fact that individuals at elevated *p* CO_2_ were smaller than control larvae, they maintained the same lipid and protein levels [43].

### Heat shock response

**Figure 4:**
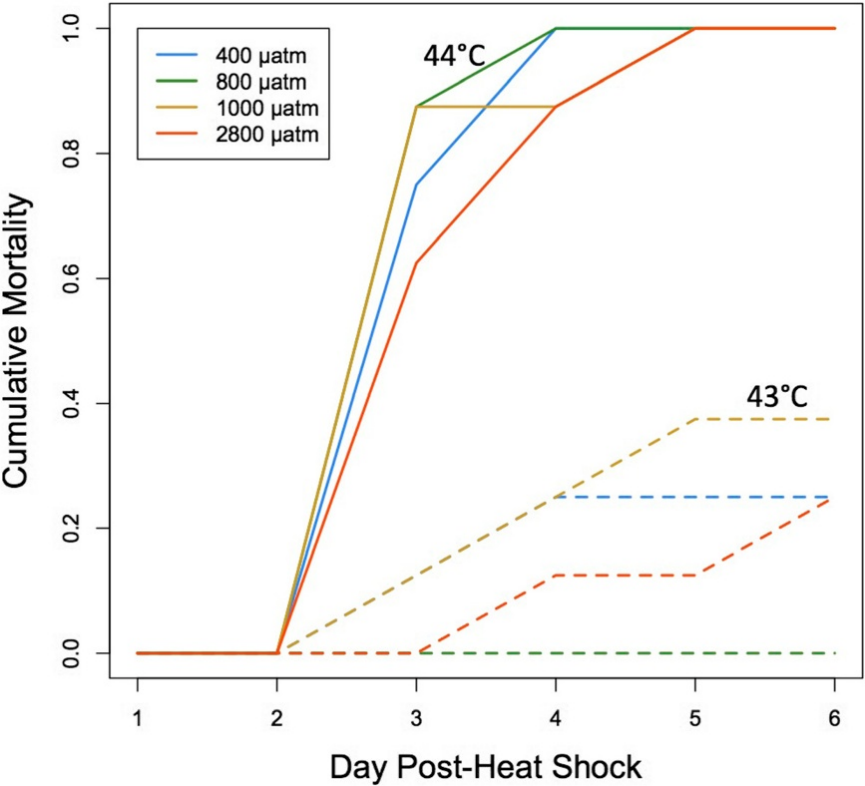
**Oyster mortality after 1 hour heat shock at 43°C (dashed lines) or 44°C (solid lines).** Mortality from heat shock did not differ among groups of oysters exposed to different *p* CO_2_ for 1 month - 400 μatm, 800 μatm, 1000 μatm, 2800 μatm.

*p* CO_2_ did not affect risk of oyster mortality in response to heat shock (p >0.05) (Figure 4) and increased temperature did increase mortality risk (z =2.073, p =0.0382). One hundred percent mortality (n =8 oysters per temperature per treatment) occurred across all three treatments by day 5 post-heat shock at the lethal temperature (44°C). No mortality occurred by day 6 post-heat shock in the 42°C group (data not shown).

Consistent with the hypothesis that elevated *p* CO_2_ impacts underlying physiology, we predicted that lower pH would depress the temperature threshold for mortality after heat exposure. The lack of influence on acute heat shock response across the four *p* CO_2_ treatments could be evidence that *p* CO_2_ has little effect on the oyster’s macro-physiological response. We are not aware of other studies that have investigated ocean acidification and acute heat stress, but there are studies that have explored moderately elevated temperatures and ocean acidification over an extended period. In *C. virginica*, elevated *p* CO_2_ did not impact the oyster’s response to elevated temperature [44]. In fact, Ivanina et al. [44] demonstrated that exposure to elevated *p* CO_2_ limited high temperature-associated mortality in *C. virginica*. It appears that extended exposure to elevated *p* CO_2_ did not mitigate oyster mortality in response to acute heat shock as it can do with long-term exposure to a moderately elevated temperature.

### Proteomics

After filtering, 700 733 peptides were considered for analysis corresponding to 1 616 proteins (Additional file 3: Table S2, Additional file 4: Table S3, Table 3). Raw data and ProteinProphet search files are available in the ProteomeXchange with identifier PXD000835. Eighty-nine percent (1 449) of proteins were annotated using the UniProt-KB/SwissProt database and 77% (1 250) of those were further categorized with Gene Ontology information (Additional file 5: Table S4).

**Table 3:** **Total number of proteins identified for each oyster across all three technical replicates with numbers of proteins for each individual technical replicate in parentheses**

Oyster (pCO _2_ – biological replicate)	Total proteins (Technical replicate #1, #2, #3)	Proteins across all 3 replicates (% of Total)
400-1	882 (869, 871, 873)	853 (96.7%)
400-2	878 (861, 863, 861)	841 (95.8%)
400-3	836 (819, 824, 819)	802 (95.9%)
400-4	867 (855, 856, 857)	839 (96.8%)
400-MechS1	861 (850, 850, 846)	832 (96.6%)
400-MechS2	815 (803, 806, 801)	788 (96.7%)
400-MechS3	854 (840, 848, 841)	826 (96.7%)
400-MechS4	862 (842, 850, 848)	825 (95.9%)
2800-1	873 (854, 858, 856)	837 (95.9%)
2800-2	924 (910, 917, 910)	894 (96.8%)
2800-3	901 (888, 884, 893)	871 (96.7%)
2800-4	874 (861, 866, 865)	849 (97.1%)
2800-MechS1	889 (877, 878, 879)	858 (96.5%)
2800-MechS2	891 (886, 876, 880)	867 (97.3%)
2800-MechS3	867 (860, 859, 856)	844 (97.3%)
2800-MechS4	941 (923, 925, 929)	905 (96.2%)

Number of proteins shared among all three technical replicates (with percentage of total) are in the third column.

**Figure 5:**
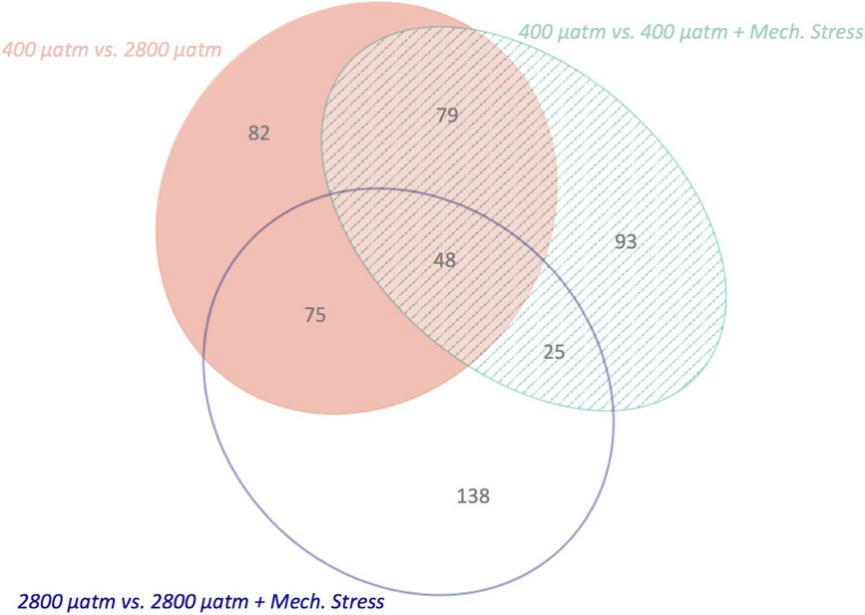
**Differentially abundant proteins among treatments.** The proteins represented by the solid ellipse were those implicated in the response to ocean acidification alone, those in the open ellipse are different in response to mechanical stress in the 2800 μatm-exposed oysters, and those in the striped ellipse changed in response to mechanical stress at 400 μatm. Numbers represent the number of proteins in each segment of the ellipses.

Pairwise comparisons were made based on gill protein expression between 1) oysters held at 400 μatm versus oysters held at 2800 μatm, 2) oysters held at 400 μatm versus oysters held at 400 μatm subjected to subsequent mechanical stress, and 3) oysters held at 2800 μatm versus oysters held at 2800 μatm subjected to subsequent mechanical stress. The magnitude of the proteomic responses in the three different between-treatment comparisons was similar (245-286 differentially abundant proteins), though many proteins were treatment specific (Figure 5). In addition, for those proteins identified as differentially abundant in more than one comparison, the directional responses of the proteins to each treatment often diverged. For example, proteins in carbohydrate metabolism and nucleotide metabolism were affected differently across treatments (Additional file 6: Figure S2). These contrasting proteomic profile variations were the most dramatic in a comparison between the responses to ocean acidification alone and to mechanical stimulation at high *p* CO_2_. All 75 differentially abundant proteins shared between these two responses had opposite relative abundances in response to stress, i.e. if a protein was found at an increased abundance in response to ocean acidification then its abundance was decreased in response to ocean acidification and mechanical stimulation (Additional file 4: Table S3, Additional file 7: Figure S3). Many differentially abundant proteins that were common across stress responses were consistent in having either increased or decreased abundance in response to stress: 96% of the proteins (n =79) in the responses to ocean acidification and to mechanical stress at low *p* CO_2_ had the same directional change and 76% of the proteins (n =25) in the responses to mechanical stress at both *p* CO_2_.

Forty-eight proteins were differentially abundant in response to all treatments and these represent the general proteomic stress response. These proteins are associated with a wide range of biological processes, including carbohydrate metabolism (malectin and α-L-fucosidase), cell adhesion (neurexin-4 and cadherin-23), cytoskeleton processes (talin-1, dynein heavy chain 3, and coactosin), mRNA processing (small nuclear ribonucleoproteins and serine/arginine-rich splicing factor 6), the immune response (allograft inflammatory factor 1), polypeptide and protein degradation (aspartyl aminopeptidase, ubiquitin carboxyl-terminal hydrolase FAF-X, and insulin-degrading enzyme), the stress response (Hsp90 co-chaperone Cdc37, universal stress proteins MSMEG_3950 and A-like), and transcription and translation (nucleolar protein 56, 60S ribosomal proteins L21 and L38, and nucleosome assembly protein 1-like 1).

#### Proteomic response to elevated *p* CO_2_alone

Ocean acidification significantly affected the underlying molecular physiology of *C. gigas* after one month of exposure. The proteomic response of oyster gill tissues under high *p* CO_2_ conditions compared to 400 μatm was characterized by increased abundance of 148 proteins and decreased abundance of 136 proteins (Figure 5A, Additional file 7: Figure S3). Proteins identified as differentially abundant include those involved in carbohydrate metabolism (i.e. α-L-fucosidase, probable β-D-xylosidase 5, succinyl-CoA ligase, and UDP-glucose 4-epimerase), cell growth (i.e. thymidine phosphorylase and tyrosine-protein kinase Yes), transcription and translation (i.e. calcium-regulated heat stable protein 1, prohibitin-2, and translational activator BCN1), response to reactive oxygen species (i.e. glutathione S-transferase Ω-1), and signaling (i.e. dual specificity mitogen-activated protein kinase kinase 1, calcium-dependent protein kinase C). Gene enrichment analysis revealed enriched proteins are associated with transcription (i.e. transcription elongation regulator 1, 5′-3′ exoribonuclease 2), cell junction organization and cell adhesion (i.e. myosin heavy chain 95F, protocadherin Fat 4), and cell proliferation and tissue development (i.e. integrin β-PS, growth arrest-specific protein 8) (Additional file 8: Table S5). Some of these processes are discussed in more detail below.

##### Carbohydrate metabolism

One clear trend identified in gill protein expression patterns in oysters exposed to elevated *p* CO_2_ was the differential abundance of proteins associated with carbohydrate metabolism. Changes in these pathways imply alterations in energetic resource use upon exposure to elevated *p* CO_2_. Protein abundance changed for a range of enzymes involved in producing glucose, perhaps revealing a shift in glucose production pathways during ocean acidification stress. Proteins in the galactose metabolism pathway (UDP-glucose 4-epimerase, UDP-N-acetylhexosamine pyrophosphorylase, and N-acetylgalactosamine kinase), which can lead to increased glucose production, were detected at higher levels in oysters from the high *p* CO_2_ treatment. Those involved in gluconeogenesis (serine-pyruvate aminotransferase) and conversion of other carbohydrates to glucose (glycogen debranching enzyme and pyruvate kinase muscle enzyme) were detected at higher levels in oysters from the low *p* CO_2_ treatment. One explanation for these changes is that during exposure to ocean acidification oysters need more glucose production in order to maintain homeostasis. Interestingly, the oysters did not mobilize glycogen stores to meet this demand as glycogen content did not differ among *p* CO_2_ treatments in whole body tissue. Glycogen stores may have been maintained because they are typically mobilized as an immediate response to a short-term stress and/or an energetic priority is placed on maintaining glycogen as an investment in fitness. In other taxa (e.g. humans [45] and yeast [46]), the general stress response usually leads to increased carbohydrate metabolism and decreased carbohydrate storage, which is similar to observations from this study. Ocean acidification appears to impact energy resource demands in the oyster, which could be of consequence during periods of immune stress or reproduction, which also require many energetic resources.

##### Lipid metabolism

The abundance of proteins involved in lipid metabolism and transport were significantly affected by exposure to ocean acidification. Proteins implicated in fatty acid metabolism (enoyl-CoA hydratase) and fatty acid transport (fatty acid-binding protein) were detected at higher levels after exposure to elevated *p* CO_2_. A protein involved in desaturation and elongation of fatty acids (NADH-cytochrome b5 reductase 1; [47]), likely for incorporation into cellular membranes, was at lower abundance in the ocean acidification-exposed oysters. Highly unsaturated fatty acids are more sensitive to oxidative damage, therefore a lower level of unsaturation may not only protect cellular membranes from reactive oxygen species (ROS) damage but may also protect molecules within the cell (reviewed in [48]). Lipid metabolism-associated genes/proteins were also expressed at higher levels in larval barnacles [49] and adult coral [50] exposed to elevated *p* CO_2_. Changes in lipid metabolism, similar to the observed changes in carbohydrate metabolism, represent a shift in how energetic resources are used during extended environmental stress.

Frequently during extended ocean acidification stress bivalves shift from metabolism of mostly carbohydrates and lipids to greater use of protein resources [51, 52]. In this study, the observed proteomic changes were not yet reflected at the level of oyster fatty acid profiles (which may also be due to tissue-specific processes), but the results suggest that with extended exposure both carbohydrate and lipid reserves would be altered by exposure to ocean acidification. For example, lipid and glycogen stores were reduced in *C. virginica* after eleven weeks at low pH [17]. The effects of changes in metabolism may already be seen at the proteomic level in *C. gigas* with decreased abundance of proteins instrumental to muscle growth (kyphoscoliosis peptidase) and muscle repair (dysferlin) as well as the immune response (lymphocyte cytosolic protein 2 and allograft inflammatory factor 1). Maintenance of a robust immune response and of healthy muscle mass are both important for the long-term fitness of oysters and ocean acidification may be affecting these processes via the reallocation of resources.

##### Oxidative metabolism

Oxidative metabolism proteins were also affected by exposure to ocean acidification conditions, imposing further changes on energy supply and perhaps increasing oxidative stress. Increased abundance of proteins cytochrome c oxidase and NADH dehydrogenase implies that oysters exposed to elevated *p* CO_2_ experienced a heightened demand for energy (i.e. increased ATP production in mitochondria). Additionally, the abundance of prohibitin increased after exposure to elevated *p* CO_2_. In mammals, greater levels of prohibitin are associated with regulating mitochondrial respiration during stress [53]. Heightened metabolism can occur in response to an ongoing stress [45] and may be an important adaptive strategy to counteract the physiological effects of elevated environmental *p* CO_2_[54]. However, this response can be species specific: increased metabolic rate was observed in *M. mercenaria*’s response to elevated *p* CO_2_ and temperature [55], while *C. gigas*’ metabolic rate was suppressed [25]. Expression of proteins and genes associated with metabolic processes are among the most common changes when invertebrates are exposed to elevated *p* CO_2_. Genes and proteins associated with metabolism were detected at lower levels in larval urchins [56], adult corals [50], larval oysters [57, 58], and larval tube worms [59]. In the mantle tissue of *C. virginica*, proteins associated with energy metabolism were expressed at higher levels after exposure to low pH [60]. Most of these other studies revealed down-regulation of metabolic pathways, while we found a shift between different pathways, perhaps indicating preferred methods of metabolism during stress. These slight differences in results may be due to life stage and tissue type or simply the increased sensitivity of using shotgun proteomics.

##### Cellular stress

Elevated abundance of antioxidant response proteins (two isoforms of glutathione S-transferase Ω-1) and cytochrome P450 1A5, which produces ROS, provided further evidence of increased oxidative metabolism. The cellular need for an antioxidant response could arise from greater ROS generation during increased metabolism [61] or ocean acidification may directly lead to oxidative stress through elevated cellular CO_2_ and H^+^[60, 62]. Nucleoside diphosphate kinase homolog 5, a protein that prevents apoptosis, was detected at lower levels at elevated *p* CO_2_. Perhaps as a result of increased oxidative stress, the apoptotic response was also elevated. Additionally, autophagy was increased through the relatively greater abundance of CDGSH iron-sulfur domain-containing protein 2. Evidence of oxidative stress at the protein/gene level [50, 51, 60] as well as apoptosis [50, 56] were also observed to increase in other invertebrates exposed to elevated *p* CO_2_. The findings in this and in previous studies support the universal occurrence of increased oxidative stress during invertebrate exposure to ocean acidification.

In the current study, a broad-scale increase in abundance of chaperones and stress response proteins was observed in response to elevated *p* CO_2_ (heat shock 70 kDa protein 4, HSP90 co-chaperone Cdc37, universal stress protein A-like protein, Hsc70-interacting protein, universal stress protein MSMEG_3950, and stress-induced-phosphoprotein 1), providing further evidence of increased cellular stress and damage during ocean acidification exposure. These results contrast with other studies, which generally report decreased abundance of cell stress proteins and genes in response to ocean acidification [49, 50, 56], however increased expression of hsp70 was reported in *C. gigas*[51]. Some of these discrepancies among studies may be due to differences in life stage, tissue type, amount of food available, or equilibrium among transcription, translation, and post-translational modifications. It is clear that elevated *p* CO_2_ impacts the cellular stress response, but likely in a manner that addresses the changing physiological needs of the organism.

##### DNA repair transcription, and translation

There were widespread changes in proteins involved in DNA repair, transcription, and translation in oysters exposed to elevated *p* CO_2_, illustrating a significant molecular response to the physiological effects of ocean acidification. At the nucleotide level, changes in protein abundance included decreased nucleoside metabolism (purine nucleoside phosphorylase) and differential abundance of proteins associated with nucleotide metabolism (nicotinamide riboside kinase 1 and adenylosuccinate lyase). Concurrently, DNA repair proteins were detected at lower levels in high *p* CO_2_-exposed oysters (poly [ADP-ribose] polymerase 3, RuvB-like 2, and X-ray repair cross-complementing protein 5) and six cell growth proteins were found to be differentially abundant (three at increased levels: hemicentin-1, thymidine phosphorylase, and tyrosine protein kinase yes). One hypothesis for this observation is that the decrease in DNA repair proteins coupled with changes in nucleotide metabolism may indicate a shift away from repairing DNA damaged by increased oxidative stress.

Abundance of proteins associated with transcription and translation would necessarily change in order to support the myriad other molecular responses to ocean acidification. The importance of these processes is reflected in the enrichment of proteins associated with transcription in the differentially abundant proteins in response to *p* CO_2_, mechanical stimulation, and both stressors at once. In ocean acidification-exposed oysters, twenty-two proteins associated with transcription and mRNA processing were differentially abundant. In terms of protein synthesis and degradation, six proteins associated with amino acid metabolism were differentially abundant: two were detected at lower levels at in 2800 μatm-exposed oysters (amidohydrolase ytcJ and betaine-homocysteine S-methyltransferase 1) and four at elevated levels (aminoacylase-1, dihydrolipoyl dehydrogenase, glutaryl-CoA dehydrogenase, and C-1-tetrahydrofolate synthase). Twenty-three proteins involved in protein synthesis and translation were differentially abundant and eleven proteins associated with protein degradation underwent significant changes in abundance, including increased levels of two that prevent protein degradation (cystatin-B, ubiquitin caroxyl-terminal hydrolase FAF-X). In larval sea urchins, genes associated with translation decreased in abundance in response to elevated *p* CO_2_[56], while in larval barnacles and adult *C. virginica* these proteins increased [49, 60]. Transcription and translation are the processes that support all other molecular and phenotypic changes. The large number of differentially abundant proteins involved in these processes illustrates their integral role in responding to environmental stress and in proteomic plasticity.

#### The effects of *p* CO_2_on the stress response

Elevated *p* CO_2_ alone may be a stressor for many species, but it may also impair the ability to respond to other physical, chemical, or biological stressors. To investigate the ability of an organism to respond to stressors under ocean acidification conditions, we additionally subjected oysters to mechanical stimulation. Qualitative differences between the proteomic responses to mechanical stimulation at 400 and 2800 μatm were reflected in the enrichment analysis, identifying differences between treatment groups at the process level.

**Figure 6:**
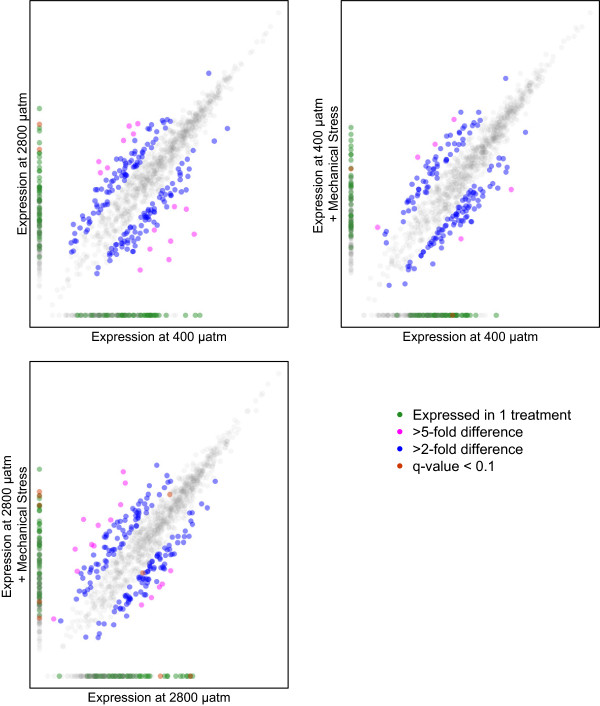
Characterization of protein abundance values (log([normalized spectral abundance factor]*10000)) for experiments designed to examine the influence of ocean acidification (A), mechanical stress at 400 μatm (B), and mechanical stress at 2800 μatm (C). Differentially abundant proteins (as defined in the methods) are represented by the bolder, colored dots and non-differentially abundant proteins appear in faint, light gray.

Two hundred forty-five proteins were differentially abundant upon mechanical stimulation at 400 μatm. One hundred seven were elevated under mechanical stress and 138 proteins decreased in abundance (Figure 6B, Additional file 7: Figure S3). The proteins that were differentially abundant included those involved in apoptosis (i.e. programmed cell death protein 5, CDGSH iron-sulfur domain-containing protein 2), carbohydrate metabolism (i.e. α-L-fucosidase, lysosomal α-mannosidase, phosphoacetylglucosamine mutase), and transcription and translation (i.e. histone deacetylase complex subunit SAP18, eukaryotic translation initiation factor 3 subunit A). Significantly enriched biological processes in the response to mechanical stress included RNA metabolism (i.e. histone deacetylase complex subunit SAP18, U1 small nuclear ribonucleoprotein A), transcription (i.e. nucleosome assembly protein 1-like 1), and gamete generation (i.e. tyrosine-protein kinase Btk29A).

One hundred forty-nine proteins were elevated and 137 proteins were detected at decreased levels when oysters held at 2800 μatm were subjected to mechanical stress (Figure 6C, Additional file 7: Figure S3). Differentially abundant proteins included those involved in response to reactive oxygen species (i.e. glutathione S-transferase μ 3 and dual oxidase 2), apoptosis (i.e. caspase-7 and engulfment cell motility protein 2), cell adhesion (i.e. protocadherin Fat 4, contactin), and signaling (i.e. E3 ubiquitin-protein ligase M|B2, prohormone-4). Polysaccharide metabolism and synthesis processes were affected by mechanical stress at elevated *p* CO_2_ as evidenced by their enrichment in this treatment, resulting from the differential abundance of proteins including glycogen debranching enzyme, glycogenin-1, UDP-glucose 6-dehydrogenase, putative glycogen [starch] synthase, lysosomal α-glucosidase, and β-hexosaminidase subunit β (Additional file 4: Table S3). Transcription was also significantly enriched in this stress response (i.e. transcription elongation regulator 1 and basic leucine zipper and W2 domain-containing protein 1).

At the metabolic pathway level, there were different proteomic responses to mechanical stimulation between the two *p* CO_2_ treatments. Specifically, in the mechanically stimulated 2800 μatm-exposed oysters, there were increased levels of proteins that convert stored glycogen into glucose (glycogen debranching enzyme, lysosomal α-glucosidase, and glycogenin-1) as well as proteins involved in gluconeogenesis (glucose-6-phosphate isomerase and serine-pyruvate aminotransferase). Rapid mobilization of glycogen stores to make glucose is a common physiological response to stress. Muscle glycogen depleted rapidly in bulls subjected to social and adrenaline stress [63] and liver glycogen similarly decreased in tilapia after confinement stress [64]. In food-stressed rainbow trout an additional handling stress caused a rapid decrease in liver glycogen and increase in plasma glucose [65]. The same exogenous stress in oysters held at low *p* CO_2_ prompted a decrease in abundance of proteins associated with carbohydrate metabolism and gluconeogenesis (neutral α-glucosidase AB, glucose-5-phosphate isomerase, and α-N-acetylgalactosaminidase) as well as increased abundance of a protein in the glycogen synthesis pathway (putative glycogen [starch] synthase), implying a relatively decreased need for readily available carbohydrate resources. At both *p* CO_2_, fewer lipid metabolism proteins were differentially abundant (four proteins at 400 μatm and three at 2800 μatm) compared to carbohydrate metabolism. Mechanical stimulation is a transient stress and carbohydrates represent a more readily available resource compared to lipids for a short-term response. The different changes observed in response to mechanical stimulation illustrate that an additional stress at high *p* CO_2_ necessitated greater resource use and a need to access energy reserves. Given the resources needed to sustain a response to an environmental stress, this increase in energetic demand could be detrimental.

In contrast to the proteomic response to mechanical stimulation at 400 μatm, oysters from the elevated *p* CO_2_ treatment had decreased levels of many proteins associated with oxidative metabolism and had a much larger antioxidant response. Four mitochondrial NADH dehydrogenases were detected at lower levels after mechanical stimulation in the high *p* CO_2_ oysters, while at low *p* CO_2_ three were detected at higher levels and one was decreased. Expression levels of cytochrome b-c1 complex, cytochrome c, and cytochrome c oxidase subunit 5B were elevated in the 2800 μatm after mechanical stress (and were not affected at low *p* CO_2_). These abundance changes suggest that at high *p* CO_2_ mechanical stress caused a decrease in electron supply from NADH to the electron transport chain (ETC) but an increase in the transfer of electrons to the terminal oxidase, while at low *p* CO_2_ more electrons entered the ETC from NADH. In marine invertebrates ROS production occurs during forward electron transport (reviewed in [66]) and a decrease in electron supply may be a physiological compensation against further ROS production. Greater oxidative stress at high versus low *p* CO_2_ was also evidenced by the increased abundance of five antioxidant response proteins upon mechanical stimulation at elevated *p* CO_2_ (glutathione S-transferase (GST) A, GST 3, GST μ 3, thioredoxin domain-containing protein 17, and dual oxidase 2) compared to just one protein at low *p* CO_2_ (GST Ω-1). These changes are further evidence that ocean acidification alters the cellular balance between energy resource supply and oxidative stress, especially upon exposure to an additional exogenous stressor.

Ocean acidification appears to significantly impact apoptotic and cellular stress responses of oysters exposed to mechanical stress (Additional file 6: Figure S2). Mechanical stimulation elicited a larger response in apoptosis pathways at 400 μatm with differential abundance of five proteins (programmed cell death protein 5, CDGSH iron-sulfur domain-containing protein 2, histidine triad nucleotide-binding protein 1, EF-hand domain-containing protein D2, and programmed cell death protein 6). At 2800 μatm only two proteins involved in apoptosis were differentially abundant: caspase-7 decreased in abundance and engulfment cell motility protein 2 increased in abundance. Five cell stress response proteins increased in abundance upon mechanical stimulation in 400 μatm-exposed oysters (protein lethal(2)essential for life, hsp90 co-chaperone Cdc37, Hsc70-interacting protein, and universal stress proteins A-like and MSMEG_3950), while four increased in the 2800 μatm-exposed (Hsp90 co-chaperone Cdc37, MAP kinase-activated protein kinase 2, heat shock 70 kDa protein 12B, and universal stress protein S||1388). These data suggest that mechanical stress is associated with apoptosis, which would be consistent with cell damage. However, apoptosis and the cellular stress response follow slightly different trajectories in response to mechanical stimulation at different *p* CO_2_ perhaps reflecting different resource availability in responding to the cellular damage inflicted by the additional stress. Changes to the normal apoptotic and stress responses could be detrimental to oysters, highlighting the importance of considering other environmental conditions when examining biological impacts of ocean acidification.

Stress from mechanical stimulation impacted abundance of proteins involved in many of the processes that were influenced by elevated *p* CO_2_, further illustrating the potential synergistic impacts of these two stressors on oyster physiology. Together these protein expression patterns indicate the complex nature of how multiple stressors influence physiology and how exposure to additional stressors (i.e. increased temperature or disease exposure) in combination with ocean acidification could have significant implications for survival and potential for adaptation.

## Conclusions

Ocean acidification is frequently portrayed as being detrimental to marine calcifiers, but its effects on invertebrates range well beyond changes to the calcification process. In this study, a wide variety of processes and responses were assessed in the Pacific oyster’s response to elevated *p* CO_2_ to better understand the physiological trade-offs that occur during this particular stress response. Shell growth was not affected by ocean acidification after one month, but elevated *p* CO_2_ did affect the integrity of the deposited shell material. Relative amounts of fatty acids and glycogen content, which are necessary for continued survival and execution of other energy-consuming processes, were also unaltered at elevated *p* CO_2_. Mortality in response to acute heat shock remained unaffected as well. The proteomic profile of *C. gigas* gill tissue was significantly altered by ocean acidification, elucidating the molecular physiological costs of elevated environmental *p* CO_2_. These changes in proteomic profile suggest that oysters experience shifts in their energy budget as they allocate resources to combat extended exposure to ocean acidification. From a proteomics perspective, ocean acidification also affected *C. gigas*’s response to mechanical stress. Shell integrity and response to a second stress become important in a dynamic environment, and in this way ocean acidification may decrease *C. gigas* fitness under chronic exposure. This research demonstrates the utility in applying proteomics technology to a study of environmental stress response in a wild population, which could lead to developing more targeted tools for studies of environmental toxicology and building models for response to *p* CO_2_ stress.

## Methods

### Ocean acidification system

This experiment was conducted in a flow-through system at the Friday Harbor Labs Ocean Acidification Environmental Laboratory, Friday Harbor, Washington, USA where oysters were exposed to *p* CO_2_ values of 400 μatm, 800 μatm, 1000 μatm, or 2800 μatm. The system and control of water chemistry has been previously described in detail [19, 24]. Briefly, incoming water was filtered (0.2 μm) and stripped of CO_2_. As the water flowed into the different treatment tanks, CO_2_-free air and CO_2_ were added back to reach set points that were continuously monitored by a DuraFET III pH probe (Honeywell, Morristown, NJ, USA). From the treatment tanks, water flowed into the eight replicate chambers for each of the four treatment levels at 57.5 mL/min. For this experiment, set points were calculated for 13°C and estimated total alkalinity (A_T_) of 2100 μmol/kg for *p* CO_2_ values of 400 μatm (pH 8.03), 800 μatm (pH 7.76), 1000 μatm (pH 7.67), and 2800 μatm (pH 7.24).

### Seawater chemistry analysis

Spectrophotometric pH was measured for all treatments 19 out of the 29 days of the experiment as described in SOP 6b [67]. On days 5, 7, 11, 14, 20, 24, and 26 spectrophotometric pH was used to measure the pH of the water inside two of the eight experimental chambers per treatment to ensure consistency with set points. Salinity was recorded with a conductivity meter (Hach sensION5, Loveland, CO, USA) and treatment temperature was verified with a Fluke 1523 thermometer (Fluke, Everett, WA, USA) whenever spectrophotometric pH was measured. Total alkalinity (A_T_) was measured using an open cell titration as described in SOP 3b [67] for the treatment reservoir water and for two chambers on days 5, 11, 20, and 26. If the A_T_ titration was not done on the day of collection, the water sample was poisoned with mercuric chloride and stored in a sealed borosilicate glass jar. CO_2_calc [68] was used to calculate calcium carbonate saturation state of aragonite and calcite, carbonate ion concentration, and *p* CO_2_ with A_T_ and pH as inputs and using the following constants: [69] for CO_2_ constants; [70] for KHSO4; total scale (mol/kg SW) for pH scale; and [71] for air-sea flux.

### Experimental design

Adult oysters (average shell length ± s.d. =51 ± 5 mm, average width =38 ± 6 mm) collected from Oyster Bay, Washington on December 29, 2011 were maintained in 3.5 L chambers (n =6 oysters per container) and acclimated for two weeks (T =13°C, pH =8). The oysters originated from the same spawning event in March 2011 from approximately 25 broodstock oysters. Oysters were fed 120,000 cells per mL per day of Shellfish Diet 1800 (Reed Mariculture, Campbell, CA, USA). Containers were cleaned every other day with freshwater to prevent fouling. At the beginning and end of the experiment, buoyant weight was measured. Relative growth rate of oyster cohorts within each treatment was calculated for buoyant weight based on [72]. For each treatment, the difference in means of natural log-transformed mass data was divided by 29 days. Analysis of variance was used to determine the main effects and interactions of time and *p* CO_2_ on buoyant weight, using the model:
1

where *bw* is the measured buoyant weight for an oyster, *t* is time point (either start or end of the experiment) and *pCO*_*2*_ is the treatment condition. Growth rate analyses were performed in R [73].

Oysters were held in one of four treatments for 29 days. At the end of the treatment period oysters were either immediately sampled (n =16), subjected to mechanical stress by centrifugation in a standard salad spinner (5min, ~100rpm) and sampled (n =8), or subjected heat shock for one hour and sampled (n =24). Centrifugation has previously been shown to stimulate a stress response in oysters as evidenced by increased circulating noradrenaline and impacts on hemocyte function [26, 27]. In this study, mechanical stimulation was used to characterize how elevated *p* CO_2_ impacts the physiological response to an additional stressor.

For sampling, a section of the posterior gill lamellae was dissected and immediately flash frozen in liquid nitrogen for protein expression analysis. The intent of the proteomics work was to focus on metabolic responses in the oyster to understand overall shifts in energy allocation. Gill, or ctenidia, tissue is frequently analyzed in oyster molecular response studies as it is metabolically active and is the interface between the oyster and its environment. Only samples held at 400 μatm (control) and 2800 μatm, both mechanically stressed and without additional stress, were considered for protein analysis. Even though 2800 μatm may seem like an extreme *p* CO_2_, oysters as nearshore animals frequently experience large, short-term fluctuations in *p* CO_2_ from both natural and physical processes [74, 75] that may impact how they respond to more prolonged low pH events. Remaining viscera from all oysters were put in a separate tube and flash frozen for fatty acid and glycogen analyses. Both shell valves were gently cleaned of remaining tissue and left to air dry for characterization of shell mechanical properties.

### Shell micromechanical properties

Micromechanical testing was conducted on left shell valves of *C. gigas* that had been exposed to 400, 1000 or 2800 μatm. All reagents, supplies and equipment for sample preparation were purchased from Allied High Tech Products, Inc. (Rancho Dominguez, CA, USA) unless otherwise stated. Micromechanical testing was conducted within the outermost 3 mm of the shell posterior, the region of the shell where growth occurs most rapidly. Although we could not definitively differentiate shell grown during the experimental exposure from pre-existing shell, observations of growth during the course of the experiment were consistent with a 3 mm deposition of new shell.

To prepare samples, shell valves were first cut across their width using a water-cooled diamond tile saw (Skilsaw, #3540), separating the anterior from the posterior portion of the shell. The posterior segment of valves (approximately 35 mm in length) was then cleaned using Micro Organic Soap and a cotton ball to remove oil and debris and mounted on a glass microscope slide using mounting wax. Slides with mounted shells were secured to the cutting arm of a low speed diamond saw (TechCut 4, cooled with proprietary cutting fluid) and the shell segment was cut longitudinally, transecting the most posterior edge. Sectioned shell valves were removed from slides, cleaned again with Micro Organic Soap, dried on a hot plate at 70°C, and mounted in epoxy resin. Mounted samples were then ground and polished on a manual grinding/polishing machine (M-Prep 5) by passing samples through a grinding series of 180, 320, 600 and 800 grit and then polishing with a 1 μm diamond suspension and finally a 0.04 μm colloidal silica suspension. Samples were cleaned with Micro Organic soap and checked under a metallurgical microscope after each step of the grinding/polishing process, and were re-polished if necessary until the surface of each sample was completely even and free of scratches. No etching of shells was observed during grinding or polishing.

Vickers microhardness tests were conducted using a microindentation hardness tester (Clark Instrument MHT-1, SUN-TEC, Novi, MI, USA) on polished shells at 0.245 N load and 5 s dwelling time. Indents were made within the bulk, foliated layer of the shell, which was easily differentiated from the prismatic and chalky regions of the shell at low magnification. Seven to eight indentations were made per sample and each indent was placed at least 45 μm away from other indents and the sample’s edges. Vickers hardness numbers (VHN) were calculated as:
2

where F is the applied load and d is the mean length of the two diagonals produced by indentation. VHN were averaged for each shell sample. Following microhardness testing, each indent was photographed at 80x magnification on a metallurgical microscope (Jenco MET-233, Portland, OR, USA) equipped with a camera (Leica EC3, Buffalo Grove, IL, USA). Photographs were used to quantify the longest crack produced by each indent, which was measured using image analysis software (Leica LAS EZ, Ver. 3.0) as the radius of a circle radiating from the center of the indent enclosing all visible cracks (Additional file 1: Figure S1). Hardness and crack radius measurements were used to calculate fracture toughness (K_c_) for each sample as described elsewhere [76, 77]:
3

where 0.0154 is a calibration constant, E is an elastic modulus (empirically determined for *C. gigas* as 73 GPa [32]), H is hardness in GPa, P is applied load in N and C is crack radius in μm.

Statistical analysis for micromechanical properties was conducted using SPSS (Ver. 19, IBM, Armonk, NY, USA). Outliers were calculated in SPSS as values greater than 1.5 times the interquartile range below or above the first or third quartile respectively, and were removed from the dataset (at most two per treatment group). Data were analyzed using one-way analysis of variance followed by post-hoc testing. Normality and equal variance was tested using a Kolmogorov–Smirnov test with Lilliefor’s correction and a Levene test, respectively. Fracture toughness data met both assumptions and a Tukey HSD post-hoc test was used. As hardness data was normally distributed but did not meet the equal variance assumption, a Welch ANOVA followed by Games-Howell post-hoc testing was applied.

### Fatty acid and glycogen analyses

Fatty acid and glycogen analyses were carried out on oysters from three *p* CO_2_ treatments (400, 800, and 2800 μatm; n =8 per treatment for 400 and 2800 μatm, n =7 for 800 μatm due to poor quality results for one sample). Whole body tissue (minus the dissected gill) was lyophilized overnight and tissues were homogenized with a pestle for use in fatty acid extractions (2.5 mg per extraction) and glycogen extractions (19.3-84.7 mg). Fatty acid extractions were performed following the protocol described in [78] except two chloroform removals were carried out. Briefly, a chloroform/methanol extraction was used to extract the organic layer from lyophilized tissue (fatty acid extraction). Transmethylation of fatty acids was accomplished by adding toluene and 1% sulfuric acid in methanol to the dried organic layer and incubating overnight at 50°C. The aqueous phase, containing fatty acid methyl esters, was then isolated using KHCO_3_ and hexane/diethyl ether. Fatty acid methyl esters were identified by running the samples on a HP 6958 gas chromatograph with an auto-sampler and flame-ionization detector using an Agilent DB-23 column (30 m, 0.25 mm diameter, 0.15 μm film) (Supelco, Bellefonte, PA, USA). Peaks were identified based on comparison of retention times with known standards. Individual amounts of fatty acids were normalized within each replicate by dividing the peak area by the sum of all fatty acid peak areas for that sample. Normalized fatty acid data were log-transformed and non-metric multidimensional scaling (NMDS) based on a Bray-Curtis dissimilarity matrix was used to compare fatty acid profiles among treatments. NMDS and ANOSIM were performed in R with the vegan package [73, 79].

Glycogen was extracted using trichloroacetic acid (TCA). Three mL of 15% TCA was added to each homogenized tissue sample and samples were vortexed and stored at 4°C for 1 hour. Samples were then centrifuged at 3,000xg for 10 minutes and 4 mL of absolute ethanol was added to 500 μl of the resulting supernatant. After an overnight incubation at 4°C, samples were centrifuged 30 minutes at 4,000xg, supernatant was removed, and the resulting glycogen pellet was dissolved in 200 μl Nanopure water. Glycogen concentration was determined using Sigma’s Glycogen Assay Kit following the manufacturer’s protocol (MAK016, Sigma-Aldrich, St. Louis, MO). Each sample was diluted 1:30 in Hydrolysis Buffer and run in triplicate. A few samples that were still too concentrated were run again diluted 1:60. Absorbances were read on a Spectra Max M2 (Molecular Devices, Sunnyvale, CA) at 570 nm using Softmax Pro v.5 software (Molecular Devices). The coefficient of variation was <20% for all samples. Absorbance measurements were blank corrected and concentrations were corrected for dilution factor and original mass of tissue used for the extraction. Differences among groups were explored using a one-way ANOVA in R [73] with *p* CO_2_ treatment as a fixed factor.

### Heat shock

Oysters from each of the *p* CO_2_ treatments of 400, 800, 1000, and 2800 μatm were subjected to acute heat shock to explore the effects of *p* CO_2_ exposure on the mechanistic response to this short-term stress. Three temperature shocks were implemented consisting of two sublethal temperatures (42° and 43°C) and one lethal temperature (44°C). The lethal heat shock temperature was previously determined for this group of oysters and is defined as the temperature at which 100% mortality occurs within one week after a one hour exposure [80]. Eight hundred milliliters of seawater was equilibrated to the correct temperature in a circulating water bath. Since oysters considerably decrease the temperature of the bath, we added a pre-heating step of 10 minutes in one beaker after which the oysters were transferred into another beaker for the full hour. After heat shock, oysters were returned to the flow through system at pH =8.03 and 13°C. Mortality was the only parameter assessed for the temperature treatment. Differences in mortality across treatments were analyzed using the Cox proportional hazards regression model [81] in R [73] with *p* CO_2_ and temperature as variables.

### Liquid chromatography and tandem mass spectrometry (LC-MS/MS)

Protein extraction and desalting were performed on gill tissue from four mechanically stressed and four control oysters held in the present day and the highest treatment levels, 400 and 2800 μatm, respectively (n =16 oysters total) as described in [82]. Each of the 16 protein samples was injected into the LC-MS/MS three times, with injections occurring in a randomized order. LC-MS/MS and data acquisition were carried out as previously described [82].

### Protein informatics analysis

Peptide tandem mass spectra were correlated to *in silico*-generated tandem mass spectra resulting from the Pacific oyster proteome (database published July 12, 2012 and accessed in August 2012) [83] using SEQUEST [84]. Using PeptideProphet from the trans-proteomic pipeline (TPP), peptides were assigned a relative score for best match to the database [84, 85]. Only peptides with a PeptideProphet probability score of at least 0.9 were considered for further analysis. Additionally, a protein was considered for analysis only if it had at least 8 spectral counts across all 48 injections (1 spectral count =1 peptide matched to that protein). Within a biological replicate, a protein was considered to have a non-zero expression value if it had at least 2 unique peptide matches.

NSAF (normalized spectral abundance factor), a metric based on spectral counting [86], was used to quantify protein expression. Total spectral counts (SpC) for each oyster were averaged across the three technical replicates. NSAF was calculated by dividing average SpC for each protein by the protein length (L) and then dividing SpC/L by the sum of all SpC/L within a biological replicate [86]. This workflow was executed in SQLShare [87] and workflow and input files are available [88].

Oyster proteins [83] were annotated by comparing sequences to the UniProt-KB/SwissProt database (http://uniprot.org) using the blastp algorithm [89] with an e-value limit of 1E-10. Based on homology with the SwissProt database, oyster proteins were further annotated with Gene Ontology (GO) and GO parent categories (GO Slim).

Fold change in protein abundance between treatment groups was found by dividing the average NSAF of four biological replicates in one treatment by the average NSAF in the other treatment. In order to statistically test for significant abundance differences, treatments were compared in a pairwise fashion (400 vs. 2800 μatm, 400 μatm vs. 400 + mechanical stress, and 2800 μatm vs. 2800 + mechanical stress) using the qvalue package in R [73, 90] with a q-value cut-off of 0.10. Use of a q-value instead of a p-value from a *t*-test allows for a multiple comparisons correction using the positive false discovery rate [91, 92]. In an effort to determine the biological processes that were influenced by altered environmental conditions, proteins were considered differentially abundant if either there was 1) a 2-fold difference in abundance between treatment groups or 2) a q-value <0.10. All proteins with a q-value <0.10 were within the 2-fold differentially abundant group except for cytochrome b-c1 complex 2, which had an increased expression of 1.5-fold in response to mechanical stress at 2800 μatm. The two caveats to these differential abundance classifications were that only proteins detected in more than one oyster were considered for fold-based analysis and only proteins expressed by all oysters within a treatment group were considered significant for the q-value cut-off. Proteins detected in only one treatment within a comparison were included in the differentially abundant protein group. These differentially abundant proteins were visualized in iPath2 using Uniprot-KB/SwissProt annotations [93, 94] for each treatment comparison. Enrichment analysis was performed on differentially abundant proteins using the Database for Annotation, Visualization, and Integrated Discovery (DAVID) v. 6.7 [95, 96] (http://david.abcc.ncifcrf.gov/). The background protein list was made from the entire sequenced gill proteome. Biological processes were considered significantly enriched if p-value <0.075. Overlaps in the responses to different stressors were explored using a Venn diagram of the proteins that were differentially abundant between treatments in eulerAPE v. 1.0 (http://www.eulerdiagrams.org/eulerAPE/).

### Availability of supporting data

All raw data accompanying this manuscript is also available through the data compilation on the biological response to ocean acidification through Ocean Acidification International Coordination Centre (http://doi.pangaea.de/10.1594/PANGAEA.837671). The data set supporting the results of this article is available in the ProteomeExchange repository, [PXD000835 http://proteomecentral.proteomexchange.org].

## Electronic supplementary material

Additional file 1: Figure S1: Representative indents made during micromechanical testing for the (A) 400 μatm and (B) 2800 μatm *p* CO_2_ treatment. The radius of a circle radiating from the center of the indent enclosing all visible cracks was used to calculate fracture toughness, a portion of which is shown for each treatment. Arrow denotes the longest crack found for each indent. Radius length is shown on the image in μm. Mean crack radius was similar between the 400 and 1000 μatm treatments. (PDF 5 MB)

Additional file 2: Table S1: Raw and normalized (proportion) fatty acid data for 8 oysters each from 400 and 2800 μatm *p* CO_2_ and 7 oysters from 800 μatm. (XLSX 66 KB)

Additional file 3: Table S2: ProteinProphet output for each technical replicate. Information for each protein includes percent coverage by sequenced peptides, total number unique peptides, total independent spectra (spectral count), and peptide sequences. (XLSX 28 MB)

Additional file 4: Table S3.: Protein abundance values (NSAF) for each oyster for the 1 616 proteins identified. Also included are average abundance values across treatments (i.e. 2800 avg NSAF is the average abundance across all four high *p* CO_2_-exposed oysters); fold change for treatment/control oysters (i.e. Fold Diff OA is [2800 avg NSAF]/[400 avg NSAF]); columns for each of the three treatment comparisons with an asterisk indicating if the protein is >5-fold higher or lower levels; SwissProt annotation, e-value, and gene description; proteins responsible for enrichment and the treatment comparisons in which they are enriched; a column indicating in which stress treatment proteins are differentially abundant (q-value <0.1). In the fold difference columns “up” signifies that the protein was only detected in oysters from the 2800 μatm treatment (versus the 400 μatm, Fold Diff OA), mechanical stress at 400 μatm treatment (versus 400 μatm, Fold Diff 400 MechS), or in the mechanical stress at 2800 μatm (versus 2800 μatm, Fold Diff 2800 MechS); “down” represents proteins that were only detected in the other treatment for each comparison. (TXT 384 KB)

Additional file 5: Table S4: *C. gigas* proteins with associated SwissProt/UniProt-KB, Gene Ontology (GO), and GO Slim annotations. (TXT 4 MB)

Additional file 6: Figure S2: Representation of key metabolic pathways that are significantly affected by ocean acidification (A), mechanical stress at low *p* CO_2_ (B), and mechanical stress at high *p* CO_2_ (C). Red lines represent pathways that are more prevalent in the stress treatments and blue lines represent those that are less prevalent. In the key, the different colored lines represent different metabolic pathways that are affected by oyster exposure to ocean acidification and/or mechanical stimulation. Figures are also available on FigShare with input files for iPath2 to allow for interactive exploration of the data [97]. (PDF 39 MB)

Additional file 7: Figure S3: Heat maps of differentially abundant proteins annotated with protein names. Protein expression values have been log-transformed. The dendrograms on the left of the heat maps represent the clustering of proteins according to expression profile. (PDF 8 MB)

Additional file 8: Table S5: Enriched biological processes for proteins >2-fold differentially abundant in the stress responses to elevated *p* CO_2_ of 2800 μatm (“OA”), mechanical stress after a one month exposure to 400 μatm (“Mech Stress 400 μatm”), and mechanical stress after a one month exposure to 2800 μtam (“Mech Stress 2800 μatm”). Table includes enriched GO term, number of proteins contributing to that GO term, p-value indicating degree of enrichment, the SwissProt accession numbers for those proteins, the fold enrichment for each GO term, and the false discovery rate (FDR). (TXT 9 KB)
