# Supplementary figures and images for: Shotgun proteomics reveals physiological response to ocean acidification in Crassostrea gigas
Source: BMC Genomics. 2014 Nov 3;15(1):951. doi: 10.1186/1471-2164-15-951 (PMC4531390; doi:10.1186/1471-2164-15-951)

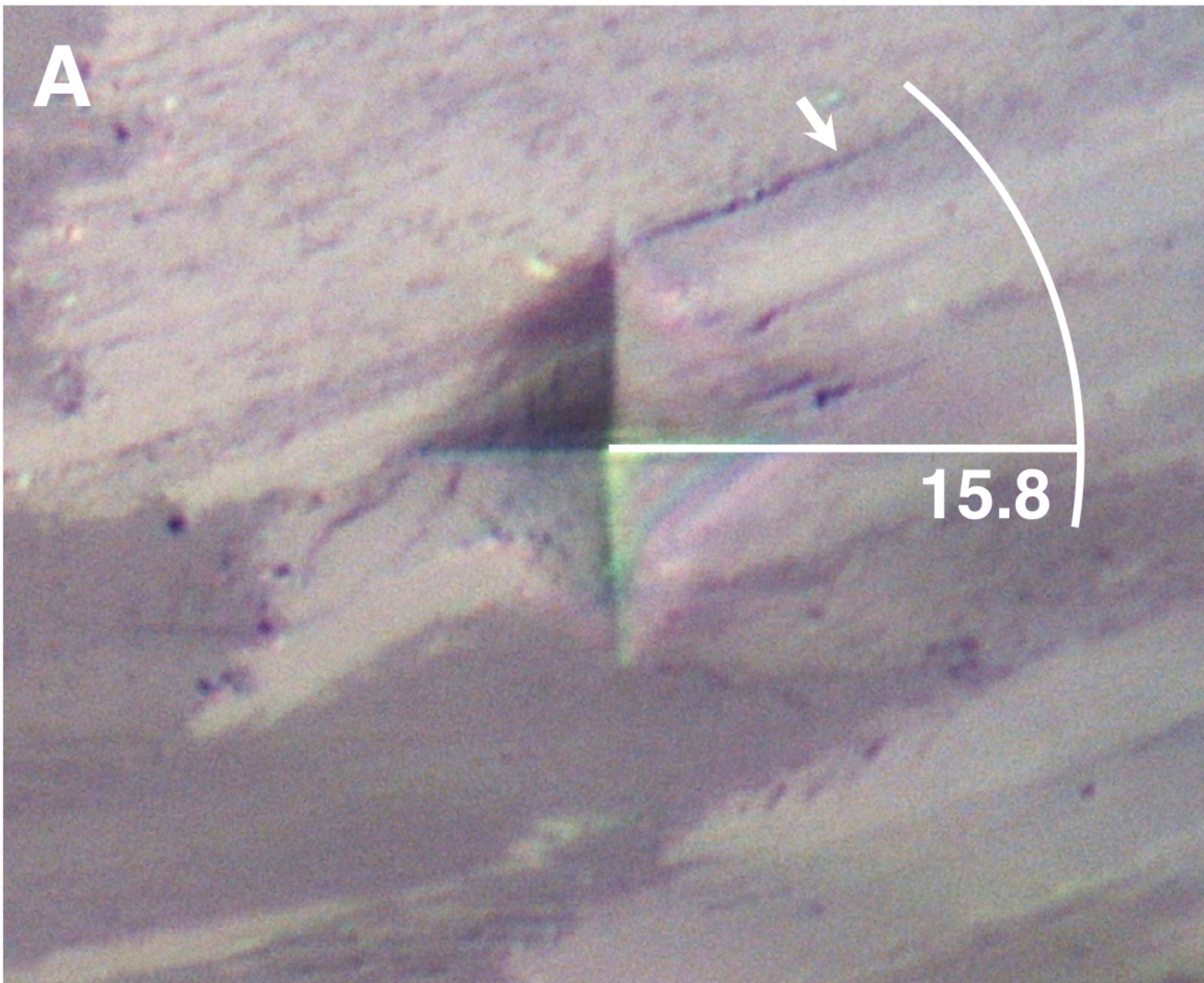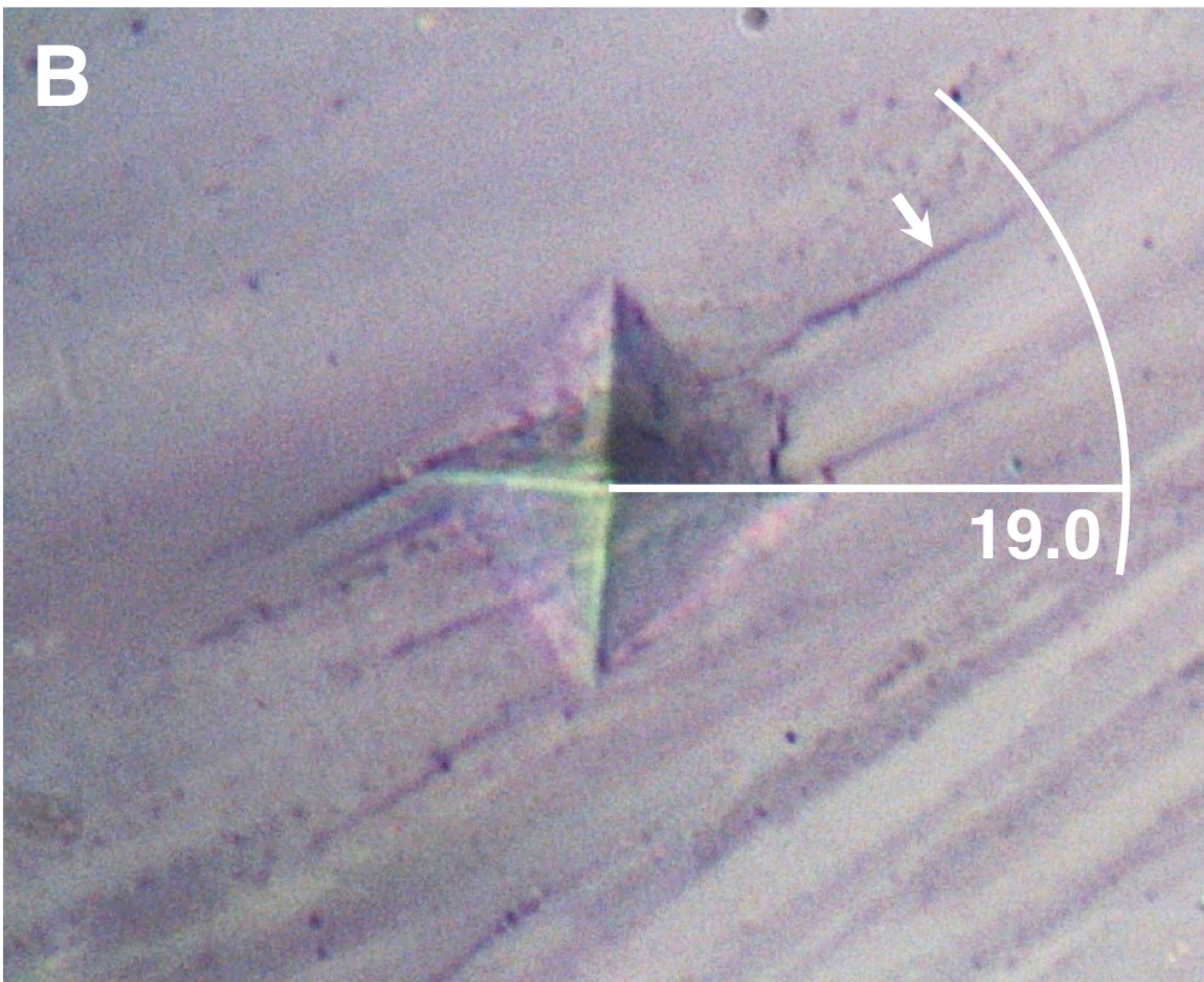

Supplement: Supplementary file 1 — Additional file 1: Figure S1: Representative indents made during micromechanical testing for the (A) 400 μatm and (B) 2800 μatm p CO2 treatment. The radius of a circle radiating from the center of the indent enclosing all visible cracks was used to calculate fracture toughness, a portion of which is shown for each treatment. Arrow denotes the longest crack found for each indent. Radius length is shown on the image in μm. Mean crack radius was similar between the 400 and 1000 μatm treatments. (PDF 5 MB) [file 12864_2014_7071_MOESM1_ESM.pdf]

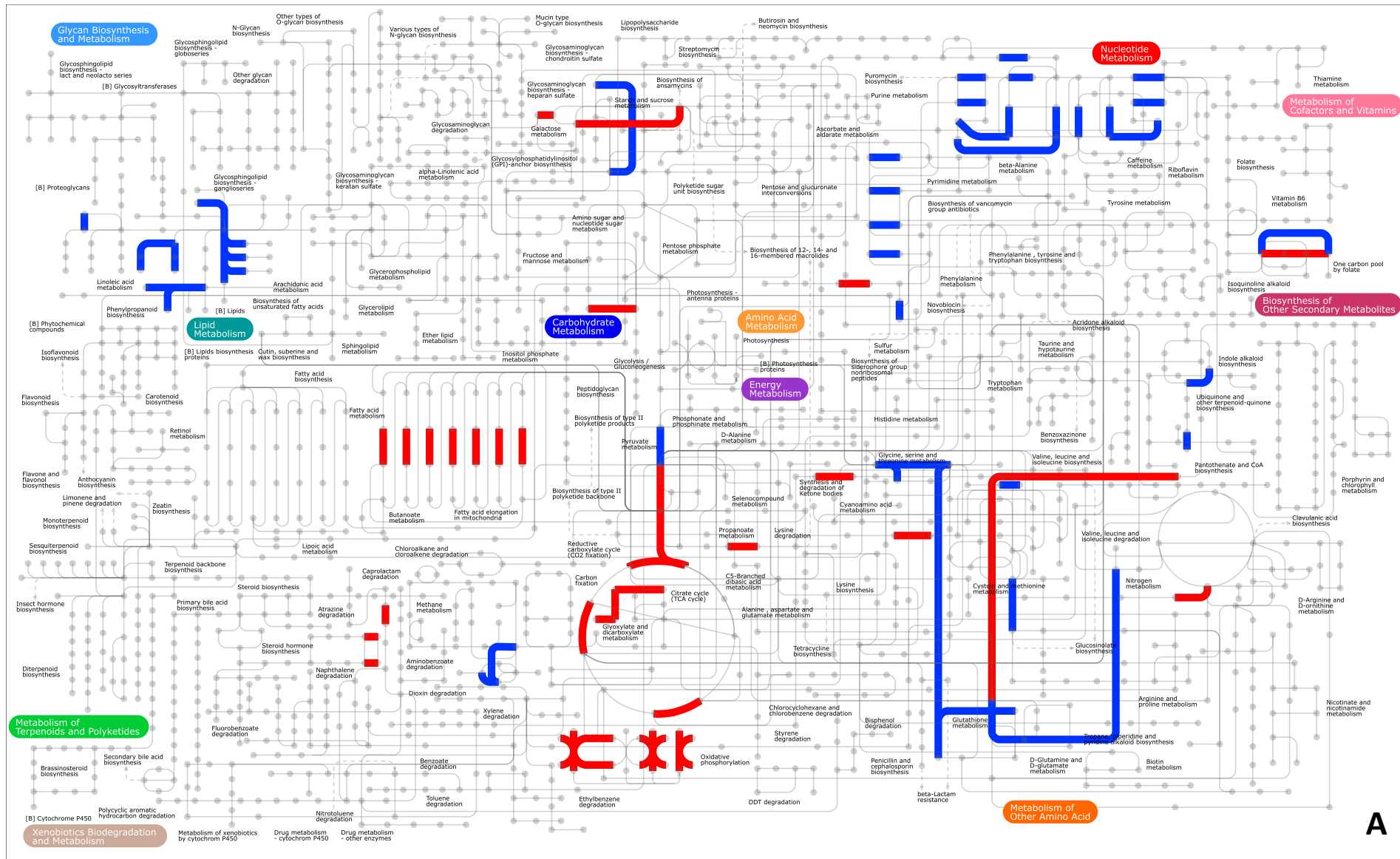

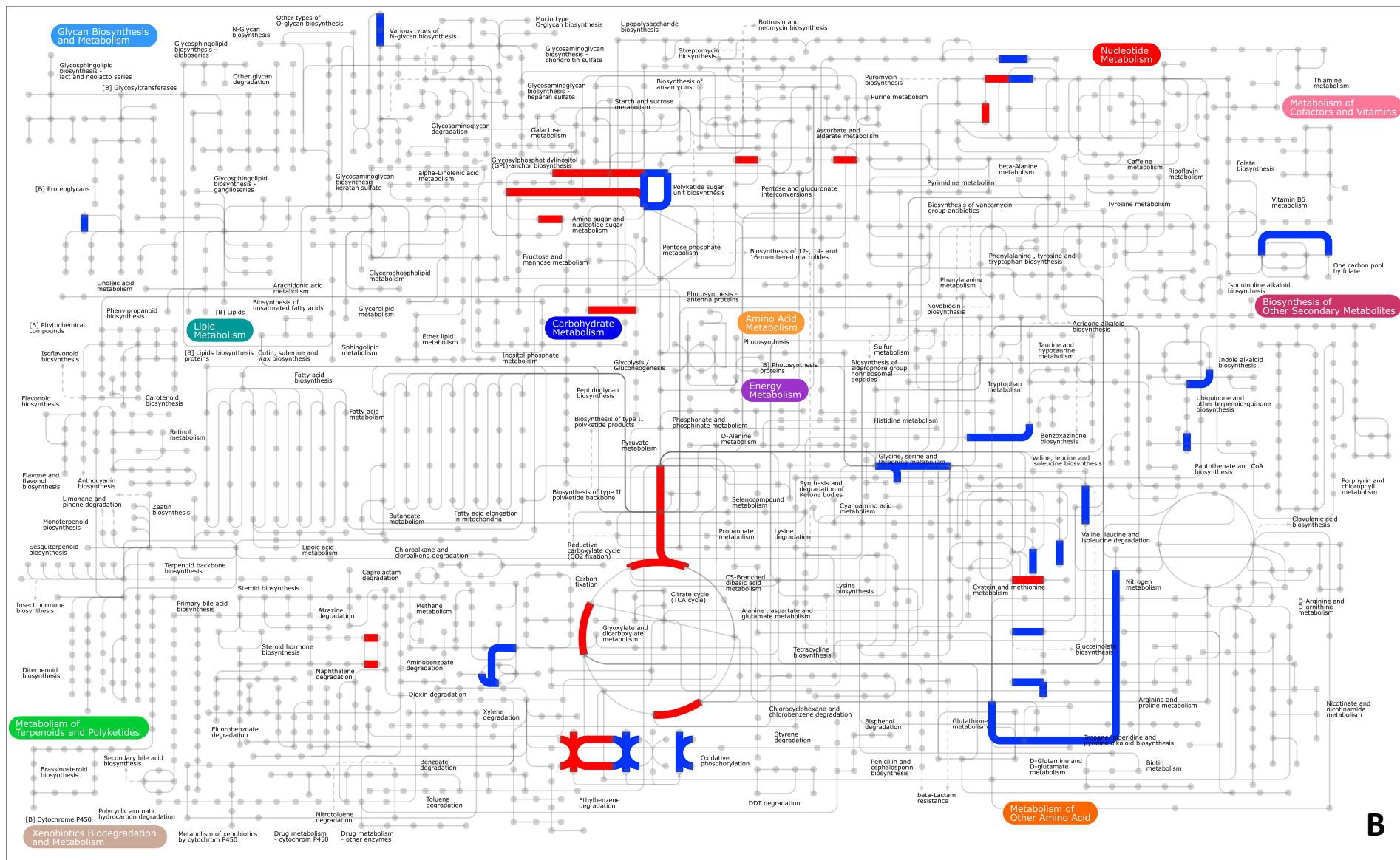

**E**

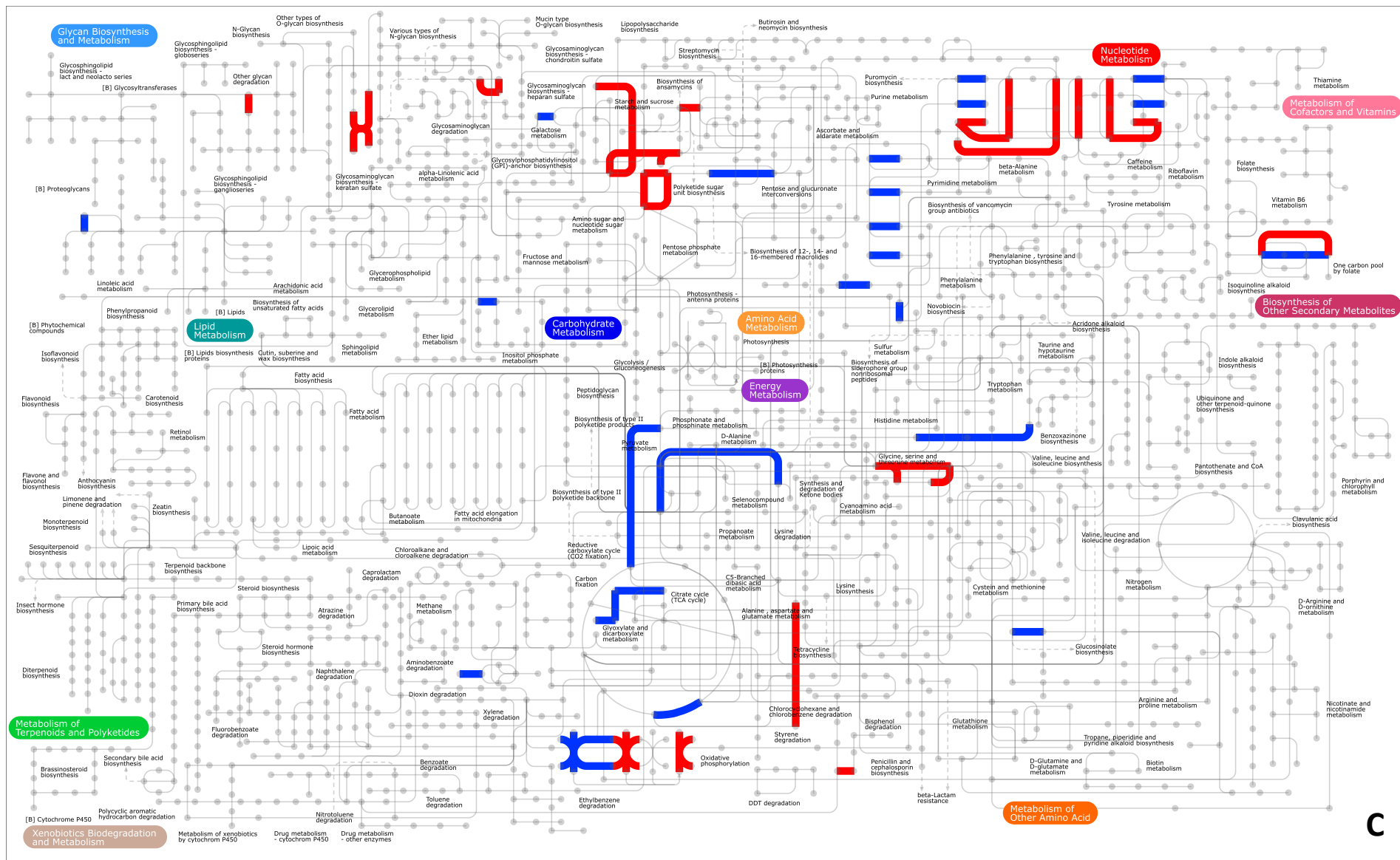

C

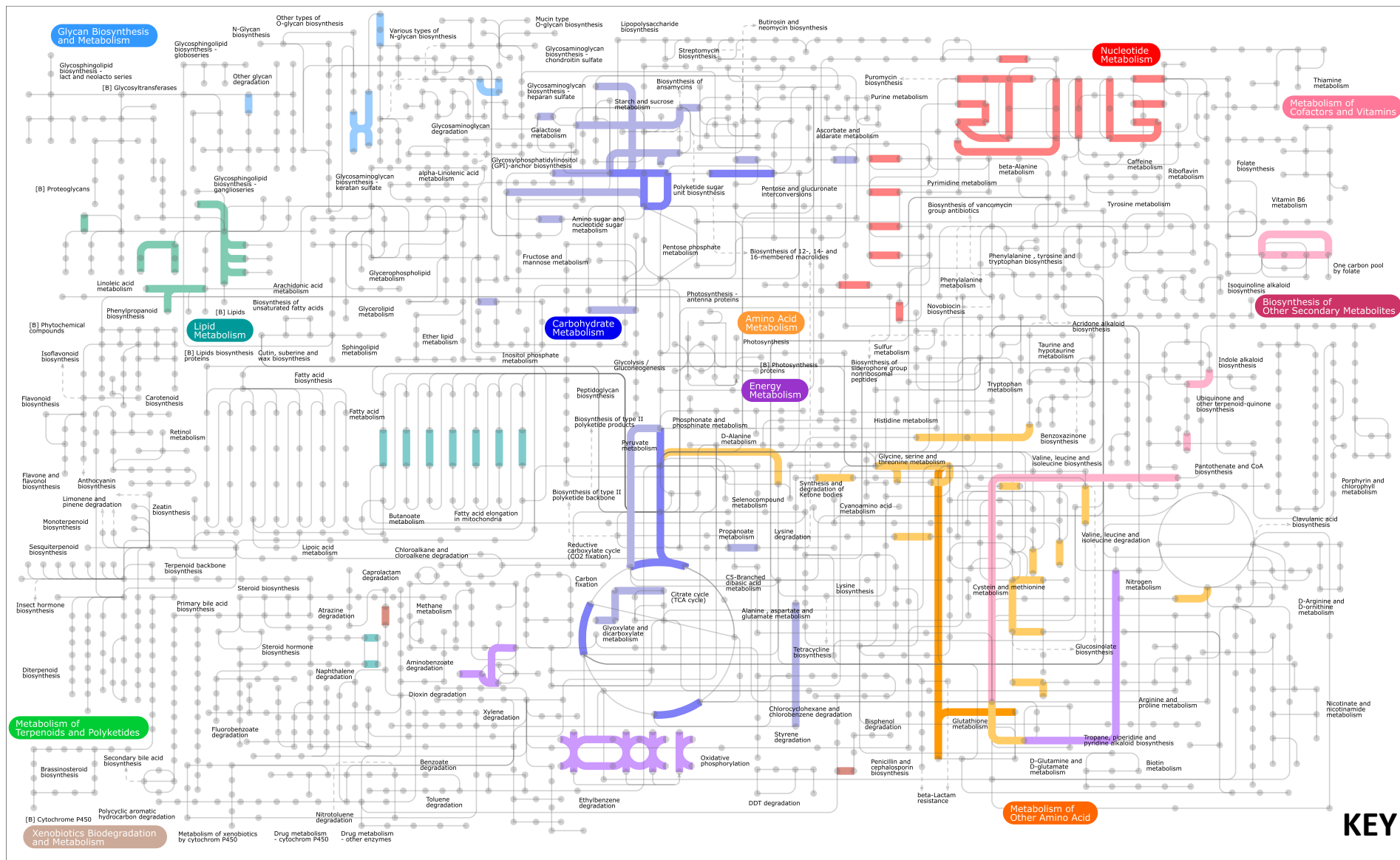

Supplement: Supplementary file 6 — Additional file 6: Figure S2: Representation of key metabolic pathways that are significantly affected by ocean acidification (A), mechanical stress at low p CO2 (B), and mechanical stress at high p CO2 (C). Red lines represent pathways that are more prevalent in the stress treatments and blue lines represent those that are less prevalent. In the key, the different colored lines represent different metabolic pathways that are affected by oyster exposure to ocean acidification and/or mechanical stimulation. Figures are also available on FigShare with input files for iPath2 to allow for interactive exploration of the data [97]. (PDF 39 MB) [file 12864_2014_7071_MOESM6_ESM.pdf]
