# Supplementary material for: Shotgun proteomics reveals physiological response to ocean acidification in Crassostrea gigas
Source: BMC Genomics. 2014 Nov 3;15(1):951. doi: 10.1186/1471-2164-15-951 (PMC4531390; doi:10.1186/1471-2164-15-951)

Ocean Acidification:
400 vs. 2800 µatm

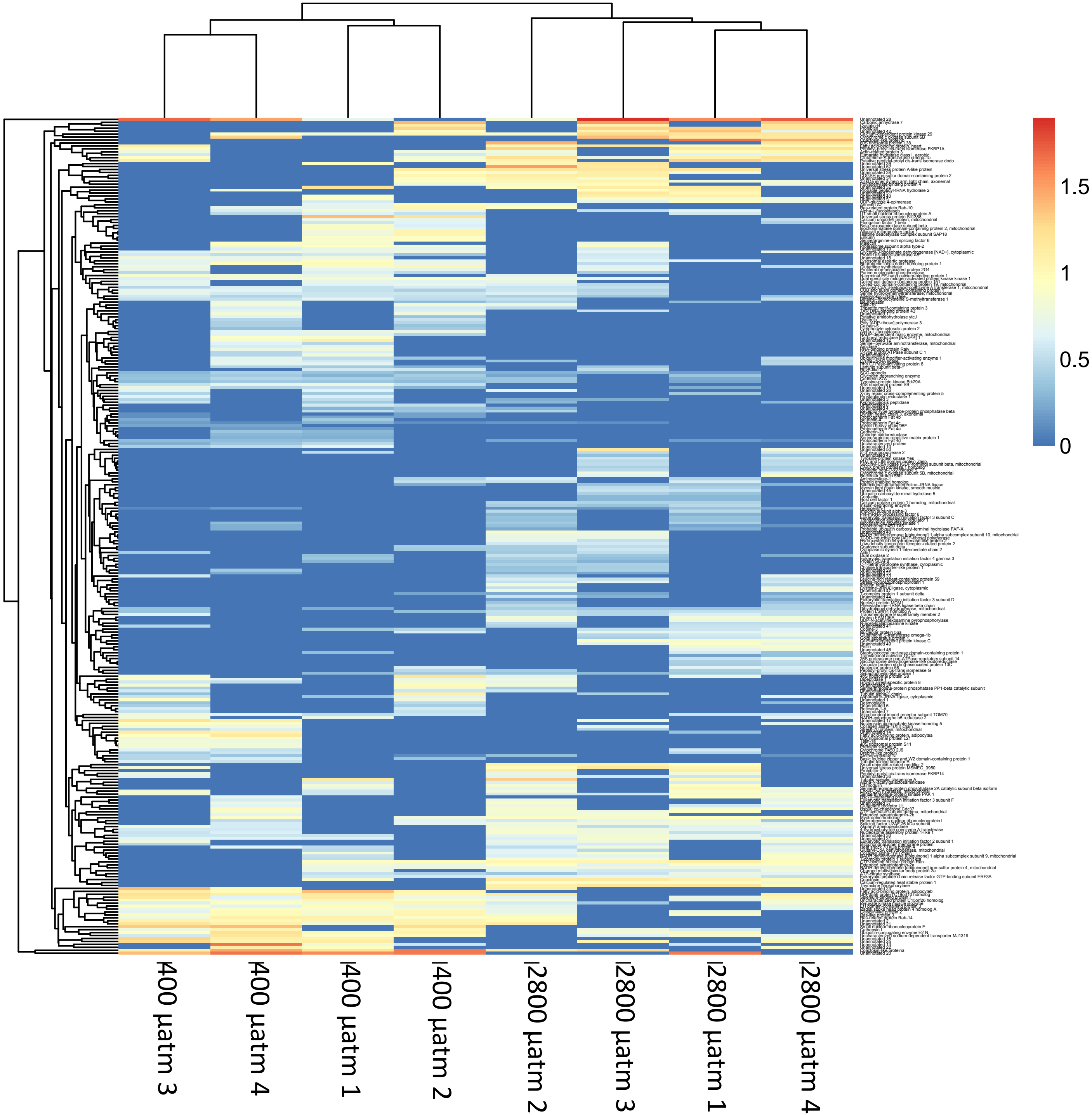

Mechanical Stress: 400 µatm vs. 400 µatm + mechanical stress

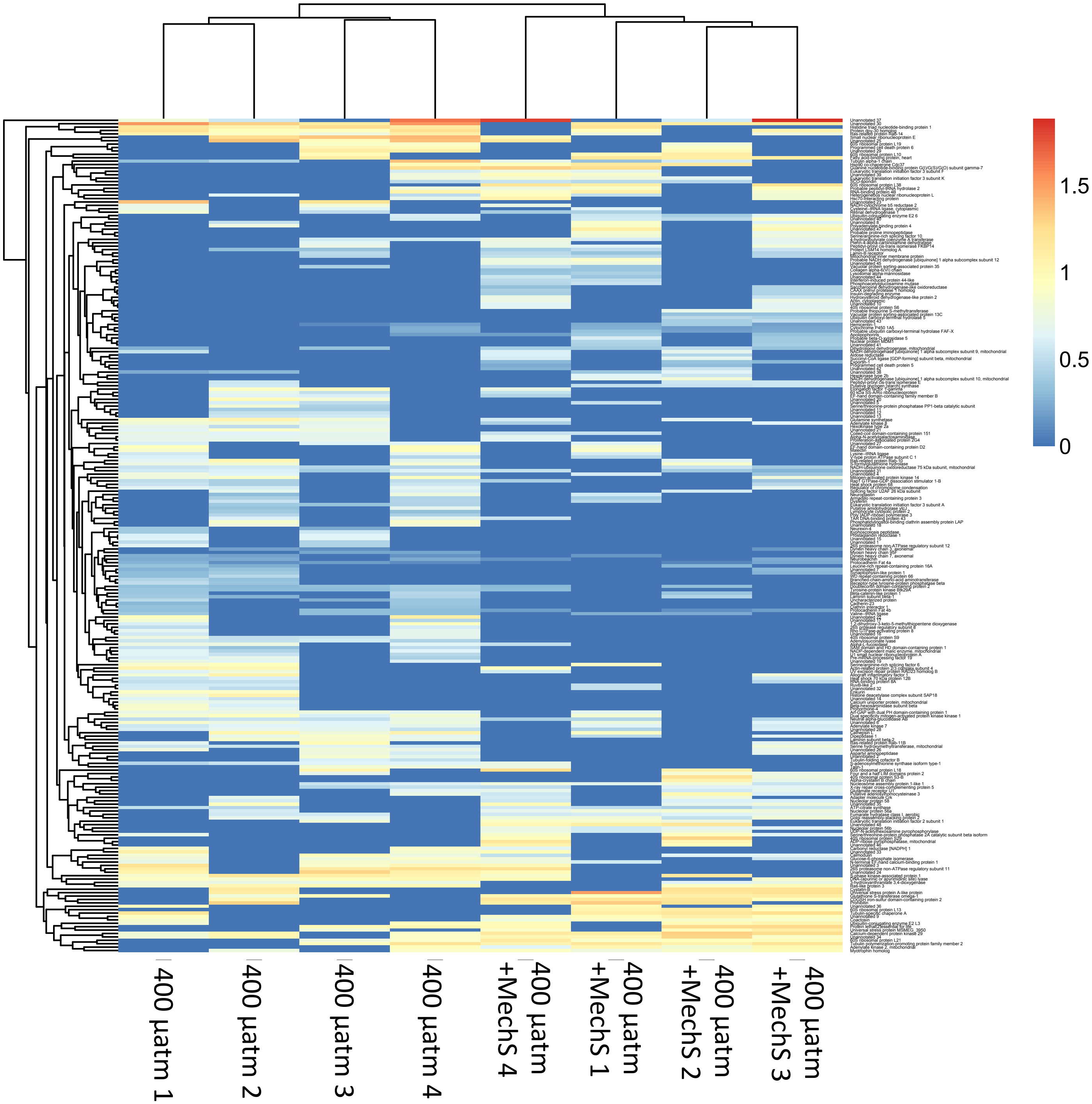

Ocean Acidification + Mechanical Stress: 2800 µatm vs. 2800 µatm + mechanical stress

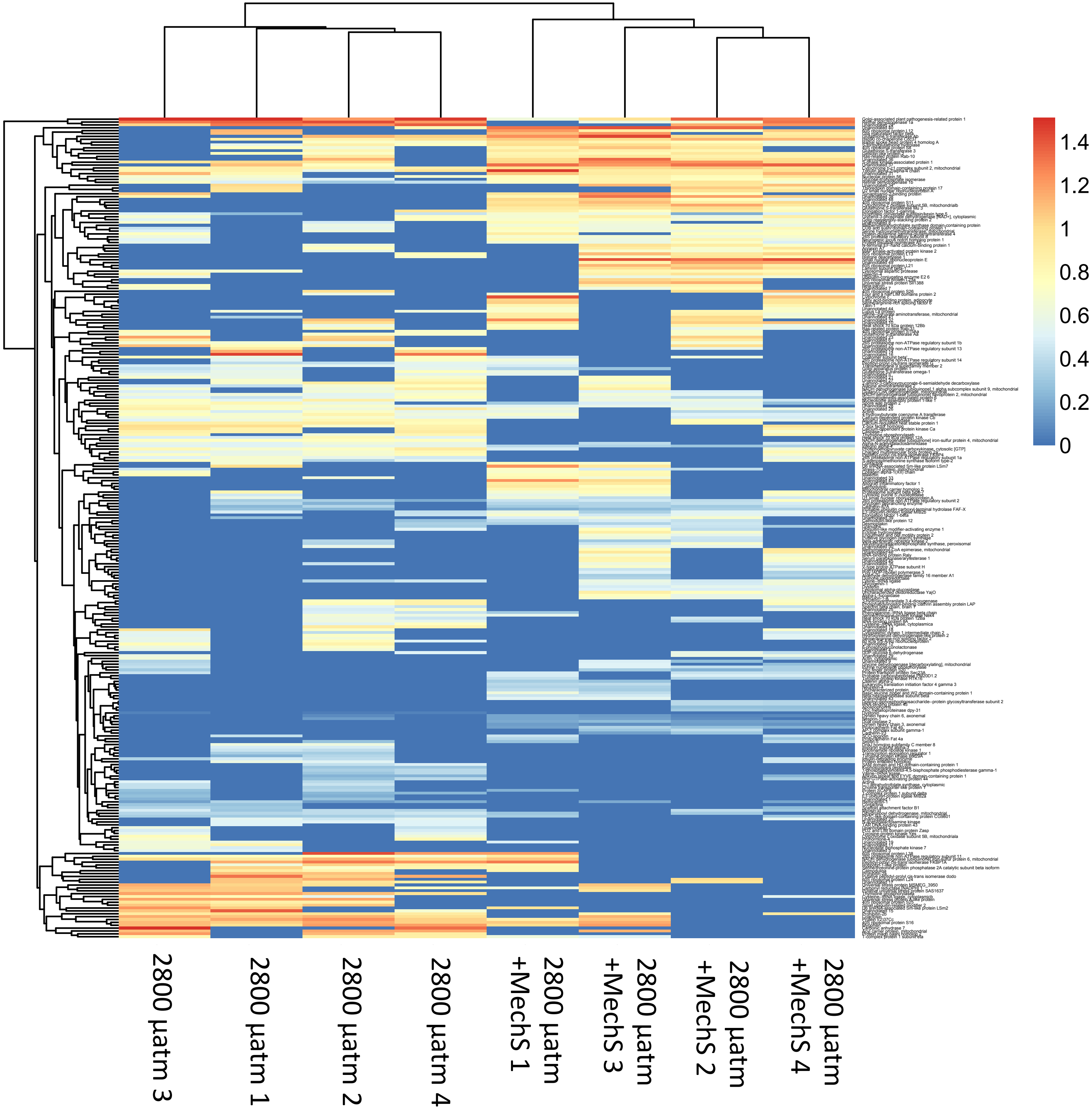

Supplement: Supplementary file 7 — Additional file 7: Figure S3: Heat maps of differentially abundant proteins annotated with protein names. Protein expression values have been log-transformed. The dendrograms on the left of the heat maps represent the clustering of proteins according to expression profile. (PDF 8 MB) [file 12864_2014_7071_MOESM7_ESM.pdf]
